# Myosin Va molecular motors manoeuvre liposome cargo through suspended actin filament intersections *in vitro*

**DOI:** 10.1038/ncomms15692

**Published:** 2017-06-01

**Authors:** Andrew T. Lombardo, Shane R. Nelson, M. Yusuf Ali, Guy G. Kennedy, Kathleen M. Trybus, Sam Walcott, David M. Warshaw

**Affiliations:** 1 Department of Molecular Physiology and Biophysics, University of Vermont, Burlington, Vermont 05405, USA; 2 Department of Mathematics, University of California, Davis, California 95616, USA

## Abstract

Intracellular cargo transport relies on myosin Va molecular motor ensembles to travel along the cell's three-dimensional (3D) highway of actin filaments. At actin filament intersections, the intersecting filament is a structural barrier to and an alternate track for directed cargo transport. Here we use 3D super-resolution fluorescence imaging to determine the directional outcome (that is, continues straight, turns or terminates) for an ∼10 motor ensemble transporting a 350 nm lipid-bound cargo that encounters a suspended 3D actin filament intersection *in vitro*. Motor–cargo complexes that interact with the intersecting filament go straight through the intersection 62% of the time, nearly twice that for turning. To explain this, we develop an *in silico* model, supported by optical trapping data, suggesting that the motors' diffusive movements on the vesicle surface and the extent of their engagement with the two intersecting actin tracks biases the motor–cargo complex on average to go straight through the intersection.

## Abstract (web summary)

Cellular cargo transported along actin filaments is faced with a directional choice at an intersection. Here the authors show that myosin Va-bound cargo prefers to go straight through the intersection, and propose a model to explain this by a tug-of-war between motors on the lipid cargo that engage the actin tracks.

The final step in the delivery of secretory vesicles such as insulin granules to the cell membrane relies on myosin Va (myoVa) molecular motors to manoeuvre their cargo through the cell's cortical actin meshwork^1^,^2^ (Fig. 1a). The actin cortex is a dense, three-dimensional (3D) cytoskeletal highway in which the plus-ends of individual actin filaments are biased towards the cell membrane, which is the direction in which myoVa travels^3^. However, this seemingly random highway, with its numerous actin filament intersections, makes efficient straight-line cargo delivery from point A to B directionally challenging (Fig. 1a). In addition, the actin cortex can act as a structural barrier to transport when cargo diameters approach the mesh size of the dense actin network^4^,^5^. Therefore, the need to define how myoVa motor ensembles deliver their cargo in a directed manner is emphasized by myoVa genetic mutations leading to mislocalized cargo such as melanosomes and endoplasmic reticulum in melanocytes and Purkinje neurons, respectively^6^, which in turn cause albinism and neurological defects in humans^7^ and the dilute mouse.

Due to the complexities of studying myoVa cargo transport in cells, investigators have characterized how individual myoVa or ensembles of these motors transport non-physiological cargo *in vitro* (for example, silica beads, DNA scaffolds) along single actin filaments^8^,^9^,^10^,^11^ or simple actin cytoskeletal models^12^. As the next step to understanding how myoVa motor ensembles meet the mechanical and directional challenges of the cell's complex 3D actin cytoskeleton, we create an *in vitro* 3D network of suspended actin filaments with numerous intersections (Fig. 1c). This network is designed to directionally challenge constitutively active myoVa motor ensembles transporting more physiologically relevant, lipid-bound vesicle cargos^9^,^13^. Motor–cargo complexes travelling along an actin filament that encounter and physically interact with a suspended intersecting filament continue straight through the intersection on the original filament it is travelling on 62% of the time. This is surprising given that the intersecting filament is both a structural barrier and an alternate track to travel on. To explain this observation, we develop an *in silico,* mechanistic model that describes the diffusive movement of motors on the vesicle surface, their engagement with the two intersecting actin tracks (Fig. 1b) and the ensuing ‘tug-of-war' between the two myoVa ensembles that eventually dictates the directional outcome at an intersection.

## Results

### 3D actin intersection and 3D myoVa transport characterization

**Figure 1:**
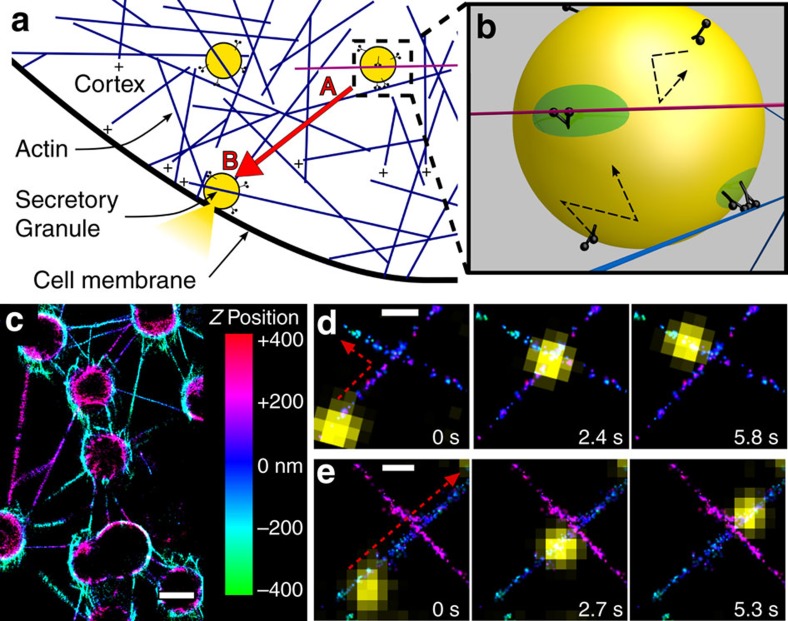
Ensembles of myoVa motors navigate lipid-bound cargo through complex 3D actin networks. (**a**) Schematic of granule (yellow) transport by myoVa ensembles through the actin cortex. Transport from A to B (red arrow) presents a number of physical and directional challenges. (**b**) Zoom in from A. Multiple-myoVa motors (black) are bound and free to diffuse (dashed arrows) on the surface of a lipid-bound cargo (yellow). One or more motors at different regions on the cargo surface (green) can simultaneously engage a single filament. In this illustration two sets of motors interact with individual actin filaments (blue, magenta) and undergo a tug-of-war to determine the direction of cargo transport. (**c**) STORM image of 3D actin network and intersections created by stringing actin between 3 μm beads; *Z*-position shown in colour. Scale bar: 2,000 nm. (**d**) Time sequence of liposome (yellow) transported by myoVa motors turning (red dashed arrow) at actin filament intersection. Actin Z-position is defined by the colour bar in B. Scale bar: 500 nm. (**e**) Liposome continuing straight through a different 3D intersection. Scale bar: 500 nm.

**Figure 2:**
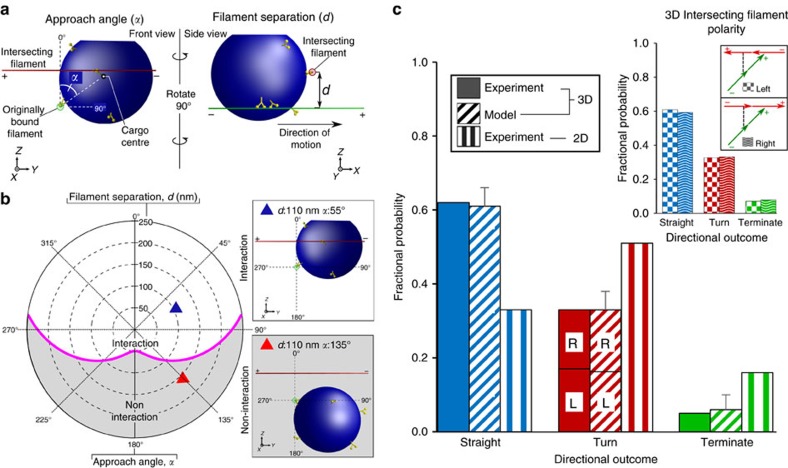
Geometry of 3D intersections and directional outcomes of motor–cargo complexes encountering actin intersections. (**a**) To scale schematic of a 3D suspended actin intersection encountered by a motor–cargo complex where the approach angle (*α*) and filament separation (*d*) are defined. An *α* of 0° has the centre of the motor–cargo complex vertically above the original filament it is travelling on, which is the same side the intersecting filament is on. *d* is the centre-to-centre distance between the two intersecting filaments at the point of the intersection. (**b**) Polar plot predicting whether or not a motor–cargo complex physically interacts with the intersecting filament as a function of *d* (0–250 nm) and *α* (0°–360°). To translate this plot into 3D spatial relations between the intersecting filaments and the motor–cargo complex, the illustration of the approach angle (*α*) in **a** is used as the point of reference. The originally bound filament on which the motor–cargo complex is travelling on comes in and out of the figure at the graph's origin. The intersecting filament is horizontally in the plane of the figure at a filament separation, *d*, above the origin with *α* defined as in **a**. The magenta line is the predicted spatial boundary at which combinations of *d* and *α* determine whether or not the motor–cargo complex can physically interact with the intersecting filament. To scale examples of interaction and non-interaction geometries are illustrated at their respective *d* and *α*. The coloured triangles for these examples are identified on the polar plot. (**c**) Fractional probability plot of motor-complex directional outcomes for experimental (solid bars, *n*=94) and modelled (slashed bars) data at 3D intersections with predicted interaction geometries. Directional outcomes at 2D experimental intersections (vertical striped bars, *n*=96) where the motor–cargo complex approached the intersecting filament on the bottom filament. Of the turning outcomes in the 3D experiments, left hand turns occurred 52% of the time with right hand turns the other 48%. For the model the left and right turn probability was equal. The model also predicts (inset) that the intersection directional outcomes are independent of the polarity of the intersecting filament. Error bars are s.d. from six simulations.

3D actin filament intersections were created by suspending actin filaments between 3 μm, poly-L-lysine-coated silica beads that adhered electrostatically to the glass surface of a microfluidic chamber (Methods section). By flowing fluorescent Alexa647-phalloidin-labelled actin filaments into the chamber through orthogonal ports, a sparse actin network was created with actin filaments running parallel to the glass surface at different heights. The height for any filament relative to the microscope's focal plane was determined by 3D super-resolution STORM imaging with 5 nm precision (Methods section)^14^,^15^. Imaged filaments (Fig. 1c–e) demonstrate uniform colours along their length, reflecting a constant height for the actin filament along its entire length. By this approach, nearly perpendicular actin filament intersections (when viewed from above in a 2D projection, Fig. 1d,e) were formed with up to 250 nm centre-to-centre filament separation at the intersection (parameter *d* in Fig. 2a). Fluorescent DiI-labelled liposomes (∼350 nm dia.), comparable in size to physiological cargos^16^, with ∼10 surface-bound myoVa motors (Supplementary Fig. 1) were then introduced into the chamber. The ability for motors to diffuse on the liposome's fluid-like surface (0.92 μm^2^ s^−1^ (ref. 17)) resulted in ensembles of motors assembling at sites of actin filament engagement, which distinguishes this study from most previous studies in which the motors' positions on the cargo were fixed and thus far from physiological^9^,^13^. The liposome transport trajectories were tracked in 3D with high spatial precision (17 nm *X*, 18 nm *Y*, 30 nm *Z*) and temporal (100 ms) resolution, using an intentionally induced optical astigmatism so that the shape of the liposome's fluorescent image defined its *Z*-position relative to the actin filament on which it travelled (Supplementary Fig. 2).

Before encountering an intersection, motor–cargo complexes travelled at velocities of 423±24 nm s^−1^ (mean±s.e.m. *n*=67) with run lengths limited by the length of the actin filaments suspended between the beads (3.7±0.2 μm, mean±s.e.m., *n*=135), since nearly all runs terminated at a bead (Fig. 1c). When compared to single-motor run lengths (∼1 μm)^8^,^17^, multiple-myoVa motors on the cargo surface must be simultaneously engaged with the actin filament (see below) to account for the nearly four-fold increase in run lengths. Motor–cargo complexes followed a spiralling path (average left-handed pitch: 2,160±40 nm, *n*=14; Supplementary Fig. 3A,B) around the suspended actin filament, allowing the motor–cargo complex to approach the intersection at any angle (parameter *α* in Fig. 2a) relative to the intersecting actin filament. Whether or not the liposome interacts with the intersecting actin filament at any point during its transit through the intersection is a simple geometric consideration, depending on the liposome diameter, its approach angle (*α*, Fig. 2a), and the filament separation (*d*, Fig. 2a). The geometries leading to whether an interaction occurs or not are graphically represented as a function of *α* and *d* on a polar plot (Fig. 2b). Given the liposome's 350 nm diameter, the motor–cargo complex will interact with the intersecting filament for all *d* between 0–225 nm as long as the motor–cargo complex approaches the intersection with an *α*<90° on either side of the original filament it is travelling on (Supplementary Fig. 4). At approach angles greater than ±90°, there are certain combinations of *α* and *d* where the motor–cargo complex can't physically interact with the intersecting filament. Knowing the importance of these spatial relationships, we characterized the directional outcomes at an intersection (that is, continued straight, turned or terminated) for those motor–cargo complexes (94 out of 103) that were geometrically predicted to interact with the intersecting filament (Fig. 2b), because those predicted not to interact with the intersecting filament (9 out of 103) went straight, as expected. For those that were spatially positioned to interact, 62% continued straight along the originally bound actin filament (Supplementary Movie 1), 33% turned (Supplementary Movie 2), while 5% either remained at the intersection or detached and terminated their runs (Fig. 2c). Interestingly, these directional outcomes are strikingly different compared with 2D intersections that are formed by adhering actin filaments directly to a glass surface.

### MyoVa transport at 2D actin intersections

To create 2D intersections where one filament lying over the top of the other could be distinguished, two separate populations of actin filaments were fluorescently labelled with either TRITC- or FITC-phalloidin and then introduced into the microfluidic chamber in sequential order. In this assay, motor–cargo complexes that approached the intersection on the bottom filament preferred turning (51%, total *n*=96; Fig. 2c, Supplementary Fig. 5D, Supplementary Movie 3) onto the upper filament at the intersection rather than continuing straight through (33%) (Fig. 2c, Supplementary Fig. 5C, Supplementary Movie 4). To go straight would have required the motor–cargo complex to first transfer onto the intersecting filament (a step up of 7 nm) and then immediately transfer back down to the original filament. In contrast, when approaching the intersection on the upper filament, the motor–cargo complex continues straight (59%, total *n*=111) with a lower turning probability (31%) as would be expected given that the lower filament is no longer a steric hindrance, coupled with the motor–cargo's approach angle being geometrically limited to ±35° due to the glass surface (Supplementary Fig. 5B,F,H). When approaching the intersection on the lower filament, changing the liposome's motor density or diameter resulted in only slight changes to the directional outcomes and, most importantly, never resulted in going straight being the predominant directional outcome (Supplementary Fig. 5E,G). Therefore, a greater probability of going straight is unique to suspended intersections, and suggests that the 3D spatial relations between the motor–cargo complex and the intersecting actin filament influence the directional outcome.

### Mechanistic model of 3D transport and laser trapping

To explain the 3D intersection data, we developed a mechanistic model that simulates the emergence and dynamic nature of motor ensembles on the liposome surface as they engage the suspended actin filaments and then mechanically interact to determine the directional outcome. In brief, the model assumes that the 350 nm vesicle and actin filaments are rigid and that 10 myoVa motors diffuse individually across the liposome's ideally fluid membrane surface (Supplementary Fig. 8; Supplementary Methods). Each motor has elastic properties both in extension (1 pN nm^−1^) and torsion (0.25 pN nm rad^−1^) via their linkage to the liposome. Once bound to an actin filament, a motor takes 36 nm forward steps that are occasionally short (31 nm) or backwards (36 nm) with all step lifetimes being dependent on the resistive load that a motor experiences^18^,^19^,^20^,^21^,^22^. Resistive loads originate from partner motors within an ensemble attempting to transport the liposome simultaneously and from motors that form within a second ensemble and engage the intersecting actin filament (Fig. 1b). The fact that a myoVa motor can take a short step under resistive load^20^,^23^ is the underlying basis for the predicted spiral trajectory (Supplementary Fig. 3B, inset). This trajectory is similar in pitch (left-handed: 2,120±200 nm, (10 simulations, mean±s.d.)) to both the experimental observations reported here (Supplementary Figs 3B and 9) and that of a single myoVa on an actin filament tightrope or multiple motors bound to a glass surface^23^,^24^. As a motor occasionally steps short on the actin helix, this motor within the ensemble places a torque on the liposome that is relieved once one of the other motors in the ensemble detaches, at which point the cargo re-centres itself over the remaining attached motors, biasing the liposome to follow a spiral trajectory. This cargo re-centring is one factor in the model that contributes to motor–cargo complexes travelling straight past an intersecting actin filament even if it presents a structural barrier (see below).

The model predicts that even though ten motors rapidly diffuse on the liposome's surface, most often three engaged motors form an ensemble at their site of engagement to the actin filament (Supplementary Fig. 10). This limited number is due to the geometric constraints dictated by the surface area of the liposome from which motors can reach the actin filament and the mechanical properties of the motors themselves. In fact, once one motor binds to actin, the spatial freedom of the liposome is restricted so that another motor joining the ensemble or engaging another filament is less probable (Fig. 4d, Supplementary Fig. 10). This three-motor ensemble prediction was experimentally confirmed by laser trapping a motor–cargo complex travelling along a single actin filament (Fig. 4a; Methods section). For ease of trapping, 500 nm silica beads were lipid coated using the identical liposome preparation so that the lipid coating has the same membrane fluidity and motor surface density. Although 43% larger in diameter, this cargo's surface area from which motors can engage the actin filament is only 23% larger than that of the 350 nm liposomes. Once engaged, the motor–cargo complex moves until stalling due to the opposing force of the trap. Three distinct populations best described the stall force distribution with peaks at 1.9±0.7, 4.1±0.6 and 5.9±0.4 pN (*n*=400). The lowest stall force population is presumably that of a single motor, given that 1.7±0.6 pN (*n*=28) stall forces were observed for experiments at limiting motor density (Fig. 4c), which agrees with the 1.8 pN forces we reported previously^19^. Therefore, as predicted by the model for the liposome size and motor density of our experiments, no more than three motors can engage an actin filament (Fig. 4c, d).

### 3D intersection geometric analysis and modelling

Finally, according to the model, the directional outcome at an intersection is the resolution of a simple ‘tug-of-war' between the motor ensembles engaged with each of the intersecting actin filaments, where the ensemble with the greater number of motors wins. In our experiments, evidence for a ‘tug-of-war' is most apparent under conditions where both the liposome diameter (>350 nm) and motor number (>25) are higher than in the present system, resulting in deformation of the liposome at a 2D intersection as the liposome is being tugged in two different directions (Supplementary Fig. 6).

**Figure 3:**
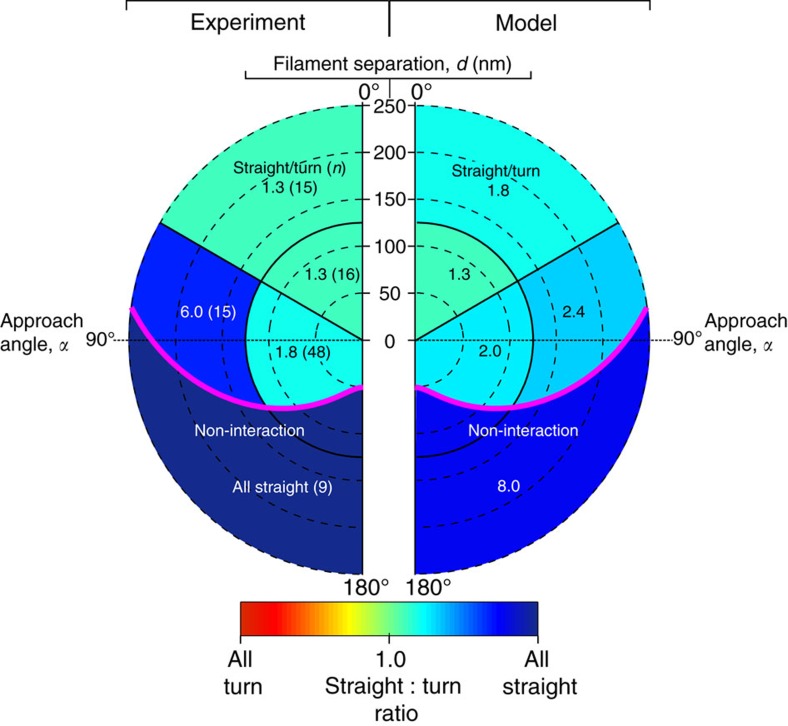
Heatmap of straight-to-turn ratio at 3D intersections plotted in polar coordinates as in Fig. 2b as a function of approach angle (*α*) and filament separation (*d*). Due to the symmetry in the system (see text), all motor–cargo approach angles between 180 and 360° were mirrored onto 0–180°(Supplementary Fig. 12A). Straight-to-turn ratio from the experimental data (left half, *n*=103) and modelled data (right half, *n*=4,000) were sorted by *α* and *d* into four interaction regimes and one non-interaction regime with the number of events for the experimental results in parentheses. The straight-to-turn ratio in each regime is colour coded (see colour bar). In both the experimental and modelled data, straight outcomes were predominant in all regimes and highest in the non-interaction regimes.

The model was used to simulate trajectories that encompassed a range of approach angles (*α*: 0°–360°) and filament separations (*d*: 50–250 nm). When we matched these to our experimental observations, the model predicts (six simulations at each actin intersection, mean±s.d.) average directional outcomes of 61±5% going straight, 33±5% that turn and 6±4% that terminate, in good agreement with the experimental results for those motor–cargo complexes that geometrically are predicted to interact with the intersecting filament (Figs 2c and 3, Supplementary Fig. 12B). Interestingly, these overall directional outcomes are independent of the plus-end orientation of the intersecting actin filament, that is, whether it faces left or right (Fig. 2c, inset). Therefore, the turning direction is determined only by the polarity of the intersecting filament and not the side of the original filament the motor–cargo complex is travelling on (that is, *α* of 0°–180°versus 180°–360°). This might explain the equal probability of turning left or right experimentally (Fig. 2c) since there should be no bias in the actin polarity when the filaments are suspended from the beads.

**Figure 4:**
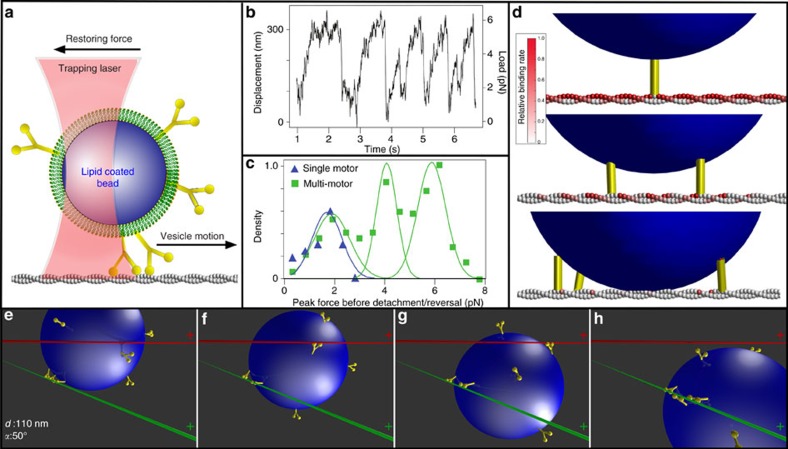
Measurement of motor–cargo complex force production and model simulations. (**a**) Schematic of laser trap experiments. (**b**) Displacement and force trace of motor–cargo complex moving against the restoring force of the laser trap (trap stiffness=0.019±0.004 pN nm^−1^) (Methods section). Saw tooth pattern arises from motors pulling the cargo until reaching a stall force (that is, plateau) and then detaching only to be repeated multiple times. (**c**) Histogram of peak force before reversal or detachment for cargo transported by multi-motors (green) and a single motor (blue). (**d**) Model predicted relative binding rate (red colour bar) for an additional myoVa motor (yellow) to available actin monomers, given 1 (top), 2 (middle) or 3 (bottom) previously bound motors. Relative rate of binding for a new motor decreases with each additionally bound motor. (**e**–**h**) Illustration of model result showing how three motors (yellow) transporting lipid-bound cargo (blue) along an actin filament (green) can go straight even when an intersecting filament (red) acts as a physical barrier at the given approach angle (*α*) and filament separation (*d*). (**f**) Single-motor engages intersecting actin, resulting in a tug-of-war between motor ensembles attached to both filaments. (**g**) Stochastic motor detachment and binding leads to cargo repositioning. (**h**) With additional repositioning, the intersecting filament no longer acts as a physical barrier, thus allowing the motors to continue straight along the original filament.

To gain additional insight into how the directional outcome may be influenced by approach angle and filament separation, we plotted the straight-to-turn ratio for the model and experimental data onto a polar plot, as in Fig. 2b. With the side of the original filament on which the motor–cargo complex approaches the intersection not affecting the directional outcome (see above, Fig. 2c, Supplementary Fig. 12A), we simplified the plot by mirroring all motor–cargo complex approach angles onto a 0°–180° range (Fig. 3). Given the large parameter space in *α* and *d* for this plot, we binned the straight-to-turn ratio into five spatial regimes. By visual inspection, in large part there is good agreement between the model predictions and experimental results with the straight-to-turn ratio being >1.0 over the entire range of *α* and *d* (Fig. 3). Therefore, regardless of the approach angle and filament separation, as long as the motor–cargo complex interacts with the intersecting filament, the probability for the complex to go straight is greater than the probability to turn. The model provides insight to this result. Prior to the intersection, the model predicts that two to three motors comprise the ensemble that is transporting the liposome. This ensemble restricts the liposome's spatial freedom as described above (Fig. 4d, Supplementary Fig. 10), which in turn limits the number of accessible actin-binding sites on the intersecting filament that a second ensemble of motors from the remaining pool of diffusing motors on the liposome surface can bind (Fig. 4d). Therefore, the likelihood that this second ensemble can develop sufficient force to win the tug-of-war and result in a turning event is low. At this point, the original ensemble will attempt to go straight but will be prevented by the intersecting filament acting as a structural barrier (Fig. 4e, Supplementary Movie 5). Thus, the motor–cargo complex hesitates at the intersection and during that period the number of motors within the original ensemble can fluctuate due to a motor's stochastic rates of attachment (∼2.4 s^−1^) and detachment (∼0.35 s^−1^). With each motor detachment and new motor attachment, the liposome spatially re-centres over the engaged motors and by doing so allows the liposome to change its approach angle (Fig. 4f,g) to the point where the intersecting filament is no longer a barrier and the motor–cargo complex is free to continue straight along the original filament (Fig. 4h). However, due to these stochastic motor number fluctuations in each ensemble, scenarios will arise in which the ensemble on the intersecting filament wins the tug-of-war, resulting in a turning event (Figs 1d, 2c and 3), which is far less frequent (Supplementary Movie 6).

## Discussion

Although the simple 3D actin network and the motor–cargo complex described here are first steps to bridging *in vitro* model systems to intracellular cargo transport, additional complexities such as denser actin networks where the motor–cargo complex could interact with multiple actin filaments simultaneously will bring both technical and modelling challenges. The addition of different motor types (for example, kinesin) to the cargo and their respective microtubule tracks to the 3D filament network are exciting future directions. Based on the present *in vitro* and *in silico* efforts, the force generating capacity of motor ensembles, fluid membranes on intracellular cargos and the geometric organization of 3D actin networks are all linked and may contribute to insuring directed myoVa intracellular cargo transport. Changes to any of these properties could then provide the cell regulatory capabilities that might allow transport to be tuned to accomplish specific cellular functions.

## Methods

### Liposome preparation and myoVa conjugation

Phospholipid liposomes of 350 nm diameter, composed of (molor ratio) 84 parts DOPC (1,2-dioleoyl-sn-glycero-3-phosphocholine), 5 parts PEG-ylated phospholipid (1,2-dioleoyl-sn-glycero-3-phosphoethanolamine-N-[methoxy(polyethylene glycol)-2000), 5 parts cholesterol, 5 parts 1,2-dioleoyl-sn-glycero-3-phosphoethanolamine-N-[4-(p-maleimidophenyl)butyramide] (MBP:PE) and 1 part carbocyanine dye DiI or DiO Cell-labelling Solution (Thermo Fisher Scientific) were created through extrusion using filter membranes^17^. In brief, the lipid mixture was mixed then dried under a nitrogen stream followed by 1 h under vacuum (Rotovap; Eppendorf). The mixture was then rehydrated to 5 mg ml^−1^ using PBS (137 mM NaCl, 2.7 mM KCl, 10 mM Na2HPO4, 2 mM N KH2PO4) at pH 7.2. Using an Avanti Mini-Extruder (Avanti Polar Lipids), the liposomes were then extruded for 20 passes using a 1 μm pore diameter filter (Whatman). Liposomes were mixed with a final concentration of 1 μM thiolated Neutravidin (SH-NaV) and incubated at room temperature for 1 h to covalently conjugate the maleimide moiety of the MBP:PE within the liposome membrane to the thiol groups of the SH-NAV^17^. After incubation, excess SH-NAV was removed by centrifugation at 420,000 *g* for 10 min. Liposomes were re-suspended in PBS (pH 7.2) and extruded for 20 passes through a membrane with pore size specific to the desired final liposome diameter, which varied between 100 and 650 nm (Supplementary Fig. 1). A double headed, heavy meromyosin myosin Va (myoVa) construct was tagged at the C-terminus with an 88-aa biotin ligase recognition sequence^25^. The myoVa was coexpressed with calcium-insensitive calmodulin light chain using a baculovirus/Sf9 cell system and was purified using affinity chromatography to a C-terminal FLAG tag on the myosin heavy chain^26^,^27^. MyoVa motors were bound to the liposome exterior surface through the C-terminal biotin on the myoVa construct binding to the SH-NaV, which is bound to the liposome^17^. Specifically, motors (3.3 μl, 500 nM) were conjugated to 350 nm DOPC liposomes (10 μl of 3.9 nM) with 6.7 μl of buffer (25 mM imidiazole, 4 mM MgCl_2_, 1 mM EGTA, 25 mM KCl, 10 mM DTT, with 1 mg ml-1 bovine serum albumin (BSA)) and incubated for 15 min at room temperature.

### Determination of liposome size and density of bound motors

Liposome size was determined by dynamic light scattering^28^. Liposome diameter was measured using a Wyatt Technology DynaPro model MSX-TX on suspended liposomes in phosphate buffered saline at pH 7.4. The measured liposome diameter was analysed using Dynamics V6 software and determined by comparing the measured diameter to a standard curve created using polystyrene beads of known size (Supplementary Fig. 1D,E). To estimate the number of myoVa motors conjugated to each liposome we used an alternative myoVa construct that in addition to its C-terminal biotin was the presence of an N-terminal Yellow Fluorescent Protein (YFP) on each motor domain (YFP-myoVa) (Supplementary Fig. 1A), and employed a fluorescence photobleaching approach described extensively in Nayak and Rutenberg^29^. To apply this method to our system, the YFP-myoVa was conjugated to the DOPC liposomes as described above. The liposomes were then imaged on a bare glass surface, using total internal reflection fluorescence (TIRF) conditions^17^. As the liposomes landed on the glass surface, the integrated intensity of each liposome was measured along with the intensity decay over time as the YFP fluorophores on the YFP-myoVa photobleached (Supplementary Fig. 1B). The intensity per fluorophore (*v*) was then calculated using equation (6) from Nayak and Rutenberg^29^,


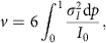


where *σ*_*I*_ is the ensemble fluorescence intensity variance across time *t*, *I*_0_ is the integrated intensity at time zero (at liposome landing) and *p* is defined as exp(−*t*/fluorescence decay rate). From the initial intensity (*I*_0_), the number of YFP-myoVa (*n*) is then calculated according to *n*=2*I*_0_/*v*. This resulted in an average of ten motors per liposome for the above mixing ratio (Supplementary Fig. 1C). By varying the motor incubation density to liposome ratio between 32 and 256:1, we confirmed that this resulted in varying numbers of motors per liposome as required for the different experimental conditions (Supplementary Fig. 1C).

### 2D actin filament intersections motility assay

The creation of 2D actin filament intersections was achieved in a flow chamber by the following steps as described previously^8^,^12^. (i) The glass surface of 20 μl flow cells were coated with N-ethyl maleimide-modified skeletal myosin in myosin buffer (0.3 M KCl, 25 mM imidazole, 1 mM EGTA, 4 mM MgCl_2_, 10 mM DTT, pH 7.4). (ii) 3 × volume actin buffer (AB) wash (25 mM imidazole, 4 mM MgCl_2_, 1 mM EGTA, 25 mM KCl, 10 mM DTT, with 3.5 mg ml^−1^ glucose, 40 μg ml^−1^ glucose oxidase, 27 μg ml^−1^ catalase, 100 μg ml^−1^ creatine phosphokinase, 1 mM creatine phosphate). (iii) 20 μl AB buffer with 1 mg ml^−1^ BSA. (iv) 3 × volume AB wash. (v) 20 μl 100 nM TRITC-phalloidin-labelled actin filaments in AB. (vi) 3 × AB wash. (vii) 20 μl 100 nM FITC-phalloidin-labelled actin filaments in AB. (viii) 3 × volume AB wash with 1 mM NaATP. Chicken skeletal actin was used for all experiments and prepared as described previously^30^.

The actin filament intersections bound to the glass surface were imaged at an exposure time of 100 ms using epifluorescence. Under intense excitation the FITC-phalloidin actin quickly photobleached, which allowed for the later imaging of DiO-labelled DOPC liposomes that share a similar excitation and emission spectra to FITC. At intersections, one can distinguish between the top and bottom filament knowing the sequential order in which the fluorescent actin filaments were introduced into the flow cell. Specifically, TRITC-phalloidin-labelled actin were introduced first followed by the FITC-phalloidin-labelled actin. After imaging the actin intersections, myoVa-bound 350 nm DiO-labelled liposomes with an average of 10 myoVa motors diluted 200 × into AB with 1 mg ml^−1^ BSA and 1 mM NaATP to a final concentration of 100 pM, were added to the flow chamber and imaged. Run length and velocities were determined from independent filaments which contained no actin–actin intersections. Run lengths were determined using a Kaplan–Meier survival estimator^31^ since a large fraction of liposomes reached the end of the actin before terminating. Velocities for these experiments were calculated by first measuring the distance travelled using the ImageJ v1.47 plug in MTrackJ^32^ and dividing by the travel time. 2D experiments as presented in Fig. 2c were performed three times with different preparations on separate days to ensure repeatability.

### 3D actin tight rope and intersection assay

Silica beads of 3 μm diameter were used to suspend single actin filaments off the glass surface. To adhere the actin to the beads, the beads were incubated under mild agitation in a solution of 400 μg ml^−1^ poly-L-lysine for a minimum of 12 h at room temperature. The beads were then washed extensively in 1 M TRIS pH 8 buffer and diluted to a final concentration of 1% solids. The beads were then passed into a custom flow chamber with orthogonal inflow ports and an internal volume of ∼30 μl and then allowed to settle to the glass surface and attach electrostatically. This was followed by a 3 × volume wash with 1 M TRIS pH 8 and then a 2 min incubation of AB–BSA buffer. Alexa 647 phalloidin-labelled actin (100 nM) was then flowed into the chamber and allowed to incubate for 2 min before being washed out. The flow helped to string actin filaments between beads. To create actin intersections, actin was introduced through the orthogonal inflow ports followed by a single AB wash. 3D STORM imaging of the actin filaments was performed in AB buffer with 1 mM NaATP and 77 μg ml^−1^ of beta-mercaptoethylamine. This buffer was identical to the buffer used for imaging liposomes with the exception of the mercaptoethylamine. MyoVa coated DiI-labelled liposomes were diluted 400 × to a final concentration of 50 pM and then very gently introduced into the flow chamber as not to disturb the suspended filaments.

### Microscopy

Fluorescent liposomes were imaged in both 2D and 3D experiments at an exposure time of 100 ms. 2D intersection experiments were performed using a customized Nikon Eclipse Ti U microscope and labelled actin and liposomes were excited using a Prior Lumen 200 epi-fluorescent lamp. The microscope was equipped with a Nikon 100 × /1.49 N.A. Plan Apo objective lens and a CCD camera (model Turbo 620 G; Stanford photonics) and image intensifier (model VS4-1845; Video Scope International). Experiments investigating the 3D helical trajectory of motor–cargo complexes moving on suspended actin filaments were performed on a modified Nikon TE-2000 inverted microscope equipped with a Nikon 100 × /1.49 N.A. Plan Apo objective lens and a Stanford Photonics XR/Turbo G intensified CCD camera, using Piper software for image acquisition. A removable 1 m focal length cylindrical lens was installed between the camera relay lens and the camera for 3D imaging, and a Custom *Z*-axis focus lock controller using optical feedback piezo stage control was used for all 3D helical trajectory experiments.

For 3D intersection assay, 3D STORM images were acquired using a Nikon N-STORM super-resolution microscope system using a 647 and 405 nm laser for excitation of Alexa 647-phalloidin-labelled actin and 3D cylindrical lens added to the light path.

### 3D image acquisition and calibration

Approximately 20,000 images were collected to generate the actin super-resolution 3D reconstruction. Minimum and maximum intensity thresholds were determined on a slide-to-slide basis, however, fluorophores with an axial ratio greater than 1.3 were eliminated from the Nikon software reconstruction. DiO-labelled liposomes navigating the actin–actin intersections were excited using a 532 nm laser. The built in NIS Nikon drift correction software and perfect focus systems were applied to ensure alignment of the liposomes and actin within the same imaging plane over the duration of any individual imaging session. Images of actin intersections and liposomes were performed by overlaying the liposome movie images to the super-resolution reconstruction using ImageJ. The 3 μm silica beads were visible in both the actin and liposome imaging channels and were used as fiducial markers to safeguard against image drift in addition to the Nikon drift correction system. The *Z*-position of actin filaments and their relationship to each other in 3D intersection experiments were determined by selecting and averaging the *Z*-positions stored in the Nikon 3D reconstruction particle table for specific regions of individual actin filaments (see more on filament separation calculation below). A *Z*-position lookup table to correct for chromatic aberrations was created using multi-colour beads adhered to the glass surface. The lookup table was generated by imaging these beads in different colour channels while stepped through different *Z*-positions (±400 nm) using the piezo stage. In addition, the four silica beads supporting the actin filament intersection were visible in all colours of the 3D experiments and served as fiducial markers. By aligning these support beads using image translation and rotation between the two colours associated with the actin filament and liposome imaging, we successfully corrected the images in *X* and *Y* as well.

### Liposome position

Raw tiff images of fluorescent liposomes were fit to a two-axis elliptical Gaussian using the Localizer program^33^ in Matlab. The normalized vertical and horizontal axis fits of the fluorescent particles were then referenced to a calibration curve derived from fits from known fluorescent particles stepped from −400 to +400 nm using a piezo stage^34^ (Supplementary Fig. 2). The difference between the index of refraction between the glass surface and the buffer results in an error in the apparent *Z*-position and was corrected by applying a rescaling factor of 0.79 to Z-localizations which was determined using the description provided in Zhuang *et al*. 2008 (ref. 35). This correction is applied by default into the Nikon NIS software. The resulting analysis provided the liposome's trajectory of *X*–*Y*–*Z* position in time. The effective accuracy of determining the liposome 3D positions was estimated by binding liposomes, via myoVa, to suspended actin filaments in AB at 0 μM ATP. This created tightly bound, stationary liposomes, which were imaged and analysed as described above. An example of these data is plotted on a 3D graph of *X,Y,Z* and the accuracy of the localization was determined by calculating the s.d. across each spatial dimension, that is, 17 nm in *X*, 18 nm in *Y* and 30 nm in *Z* (Supplementary Fig. 2C,D). Because the measurement conditions of Supplementary Fig. 2 matched the conditions of the liposome tracking experiments, the reported liposome localization precision in the 0 μM ATP condition represents the uncertainty due to the combined movements of the vesicle attached to the suspended actin filament through the myosin motors and the filament itself.

For experiments investigating the 3D helical trajectory, all liposome *X*–*Y*–*Z* position versus time trajectories were transformed so that the direction of motion was identically aligned to the *Y* direction. All trajectories were plotted in 3D space using the PyMOL Molecular Graphics System, Version 1.7 (Schrödinger, LLC) as in Supplementary Fig. 3. To determine if the trajectories were helical or non-helical, the *X-* and *Z*-positions in time were then fit to a sinusoidal function of the general equation *G*(*t*)=*A* × sin(*ω* × *t*+*ϕ*), where *G*(*t*) is either the *X*- or *Z*-position as a function of time *t*, *A* is Amplitude, *ω* is 2 × π × frequency and *ϕ* is the phase. *Z*-fits were constrained to have an amplitude between 50 and 500 nm and a frequency between 0.001 and 10 s^−1^ as demanded by the limitations of the imaging conditions, geometric constraints and known properties of myoVa. Goodness of fit was determined by root mean squared deviation. Trajectories, which failed to converge under these conditions, were considered to be non-helical. The helical trajectory was determined to be left-handed or right-handed by comparing the relative phase shift between the sinusoidal *X-* and *Z*-position fits. In addition, the 3D PyMOL visualization served as a validity check for this analysis as the helical trajectories and handedness were visually apparent as compared to the non-helical trajectories which appeared linear when plotted. Velocities along the *Y*-axis for helically rotating particles were calculated using a linear fit to a graph of *Y*-position versus time.

### Directional outcomes at actin filament intersections

In both 2D- and 3D-intersection experiments, intersection directional outcomes were determined by overlaying the movies of myoVa-bound liposomes over the actin tracks. A trajectory terminated if the liposome diffused away from the actin filaments while the liposome was positioned over the intersection's spatial centre in the 2D experiments or in the 3D experiments when the 3D spatial position of the liposome was such that it would be physically interacting with the intersecting filament. ‘Straight' events were defined by the liposome exiting the intersection on the originally bound filament and a ‘turn' event when exiting on the intersecting filament. Turns were determined to be ‘left' or ‘right' by defining the intersection from the perspective of a viewer looking top down so that the intersection formed the cardinal directions of a compass. In this analogy, all trajectories were aligned so vesicles would start at ‘South' and move ‘North' towards the intersection. Turning right at the intersection would be a turn toward ‘East'; turning left would be a turn toward ‘West'. 3D experiments as presented in Fig. 2c were performed 23 times with different preparations on separate days to ensure repeatability.

### Liposome approach angle and intersecting filament separation

For 3D intersection experiments, the approach angle was determined by using the Pythagorean relationship between the measured 3D positions of the originally bound actin filament and the liposome for the 10 frames immediately before reaching the actin intersection's centre (Fig. 2a). Approach angles (*α*) were calculated relative to the actin filament intersection. A 0° approach angle represented a fully vertical liposome travelling on the same side of the actin filament as the intersecting actin filament, while 180° represented a liposome travelling on the opposite side of the actin filament away from the intersecting actin filament (Fig. 2a). A propagation of error calculation was performed on the approach angle using the uncertainties on the actin filament position, liposome position and radius of the liposome. The error was calculated to be a function across the possible values of approach angle from 0 to 180°. The maximum error was calculated to be ±11.6° at approach angles of 0 and 180° and the minimum to be ±3.5° at an approach angle of 90°. The average error across all angles was ±7.9°. Filament separation (*d*) represented the centre-to-centre perpendicular distance between the two intersecting actin filaments at the point of the intersection (Fig. 2a). The mean *Z*-position of each filament comprising the intersection was determined from the STORM 3D fluorophore localizations within region of interest volumes on both sides of the intersection (see Supplementary Fig. 14 and legend for additional details). Once obtained, the filament separation at the intersection was simply the difference between the two filaments' *Z*-positions. The reported directional outcomes data are only for intersections that had a filament separation <250 nm, as larger separations could lead to spatial geometries beyond our calibrated *Z*-position range.

### Non-interaction and interaction geometries

Supplementary Fig. 4 describes the spatial geometries that predict a physical interaction between the motor–cargo complex and the intersecting filament in relation to filament separation (*d*) and approach angle (*α*). As the liposome's approach angle increases from 0° (travelling on the same side of the actin filament as the intersecting filament) to an approach angle of 180° (travelling on the opposite side), the centre of the liposome follows an arc as the cargo rotates around the originally bound filament. A sinusoidal function is needed to predict the maximum filament separation at each approach angle, which would allow the motor–cargo complex to still interact with the intersecting filament (Supplementary Fig. 4).


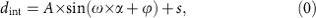


where *d*_int_ is the maximum calculated filament separation in nanometres that an interaction would still be geometrically possible, *A* is the amplitude in nm, *ω* is the angular frequency in radians per seconds, *α* is the approach angle, *ϕ* is the phase in radians and *s* is a vertical offset for the length of a myoVa. The equation calculates *d*_int_ as a function of approach angle, *α*, using the vesicle diameter (350 nm Supplementary Fig. 1), and the estimated reach of a surface-bound motor to an actin filament (that is, length of myoVa HMM (50 nm))^36^ to define the other constant variables. For example, at approach angle of 0 the maximum filament separation, *d*_int_, would be 450 nm:50 nm for the myoVa bound to the original filament, 350 nm diameter of the cargo and 50 nm for the reach of a freely diffusing motor on the surface of the cargo (Supplementary Fig. 4A).

### Heat map of 3D intersection outcomes

Figure 3 superimposes the directional outcome data at an intersection onto the polar plot (Fig. 2b) that identifies the spatial geometries of the motor–cargo complex relative to the intersecting actin filament (that is, approach angle (*α*) and filament separation (*d*)) that predict the potential for a motor–cargo complex interacting with the intersecting filament. Specifically, a straight-to-turn ratio (Fig. 3) was calculated by dividing the total number of straight events by the total number of turns observed within each of five geometrically defined regimes. The demarcation for four of these regimes attempted to create relatively equal spatial zones for geometries that resulted in a motor–cargo complex interacting with the intersecting filament. For these the boundaries were an *α* of 60° and a filament separation of 125 nm.

### Force development measurement by laser trapping

The liposome preparation used in all other experiments was first completed in full then the following additional steps were taken which were modified to our purposes from Mornet *et al*.^37^ and Bayerl and Bloom^38^ to produce lipid-coated silica beads used in the optical trap. Liposomes were diluted 3 × into a buffer of 10 mM HEPES, 150 mM NaCl. The liposomes were sonicated with a model 550 Fischer scientific sonic dismembranator using a 1/8′ probe tip in 0.5 s on–off pulses for 10 min while being protected from temperature increases by suspension in an ice bath. The liposomes were then centrifuged at 5,000 *g* for 10 min to remove any metal shards from probe tip sonication. An aliquot of 50 μl of 500 nm diameter silica beads, at a concentration of 2% solids (Duke Standard) was rinsed with (1 ml) methanol, then vacuum dried and then re-suspended into 200 μl of 10 mM HEPES, 150 mM NaCl buffer. The liposomes were incubated at 60 °C for 2 min then mixed with the silica beads. The bead–liposome mixture was vortexed then shaken for 1 h to ensure adsorption of the lipid bilayer onto the glass beads. Lipid-coated silica beads were separated from free liposomes by centrifugation at 5,000 *g* for 3 min, removing the supernatant and then re-suspending the pellet in 200 μl of PBS pH 7.4. This was repeated for a total of three washes before final suspension of lipid-coated silica beads in 200 μl of PBS (pH 7.4). Liposomes were conjugated with myoVa in the same manner and concentration as described above, with the exception of the limiting motor condition used to define unitary stall force of a single motor.

Laser trap data were captured by a custom built laser trap system that is also equipped for simultaneous TIRF imaging^20^. The system is based on a Nikon TE2000 inverted microscope equipped with a 100 × PlanApo 1.49 N.A. objective, and a 2.5 W 1,064 nm trapping laser (Spectra Physics, Santa Clara, CA), which can be steered by an acoustic optical deflector (AOD, Neos Technologies,Inc.). The brightfield image of the trapped bead is projected onto a quadrant photodiode detector (QPD), providing bead *X*- and *Y*-positions, which were filtered to 4 kHz and recorded following analogue to digital (A/D) conversion. The system provides simultaneous fluorescence imaging using through-the-objective TIRF microscopy with a 488 nm argon ion laser for excitation (Spectra Physics, Santa Clara, CA). The fluorescence image was projected onto an intensified CCD camera (XR-Mega S30; Stanford Photonics, Stanford, CA).

Experimental buffers and conditions were identical to those described above under the heading ‘2D Intersections Motility Assay.' In brief, after flow cell preparation, individual beads were visually identified and trapped in solution (AB with 1 mM NaATP) above the slide surface. Calibration of the trap stiffness, particle size and detector response was performed for each bead according to the procedure described previously^39^. For this calibration, the trapped bead's location is oscillated using the AOD (10 Hz, 113 nm amplitude) and a power spectrum is calculated for the resulting QPD signal (Supplementary Fig. 7A). Using the ‘spike' in the power spectrum at 10 Hz, the detector response calibration is calculated according to equations (12–14) from the study by Tolic-Norrelykke *et al*.^39^. Subsequently, the trap stiffness is calculated according to equation (15)^39^, with values from fitting equation (9)^39^ to the measured power spectrum. Trap stiffnesses ranged from 0.013 to 0.024 pN nm^−1^ (mean=0.019±0.004 pN nm^−1^). The linear range of the QPD detector was determined by steering a trapped bead (using the AOD) across the full range of the detector. The resulting scan (Supplementary Fig. 7B) demonstrates that the detector response is linear with the bead's position over a range of 500 nm. After calibration of detector response and trap stiffness, each bead was then lowered to a surface-immobilized TRITC-labelled actin filament (Fig. 4a). Once lowered, repeated displacements of the bead from the trap centre were observed with the displacement direction an indication of the actin polarity. To ensure that the bead's full range of motion remained within the linear range of the QPD detector, the trap centre was slowly repositioned towards the actin's presumptive ‘pointed' end, using the AOD, so as to maximize utilization of the linear range of the QPD. The largest recorded bead displacement was 386 nm from the trap centre, which is within the detector's linear range. Peak forces generated by the myoVa motors against the stiffness of the trap were identified as the maximal force within a 250 ms-wide sliding window, such that the velocity over the first half of the window (125 ms) was >50 and <−50 nm s^−1^ in the subsequent 125 ms (Fig. 4b).

Under multiple motor conditions, 400 peak forces were identified from seven beads (representing three separate bead preparations). Under limiting motor conditions, 28 peak forces were identified from three separate beads (representing three separate bead preparations).

### Code availability

The code that support the findings of this study are available from the corresponding author upon request.

### Data availability

The data that support the findings of this study are available within the article and from the corresponding author upon request.

## Additional information

**How to cite this article:** Lombardo, A. T. *et al*. Myosin Va molecular motors manoeuvre liposome cargo through suspended actin filament intersections *in vitro*. *Nat. Commun.* **8**, 15692 doi: 10.1038/ncomms15692 (2017).

**Publisher's note:** Springer Nature remains neutral with regard to jurisdictional claims in published maps and institutional affiliations.

## Supplementary Material

Supplementary Information Supplementary Figures, Supplementary Table, Supplementary Methods and Supplementary References

Supplementary Movie 1 Fluorescent movie of MyoVa cargo complex continuing straight at a 3D actin intersection. Cargo complex yellow; actin colored by Z-position defined in Fig. 1C. 3X real time. Scale 500nm.

Supplementary Movie 2 Fluorescent movie of MyoVa cargo complex turning at a 3D actin intersection. Cargo complex yellow; actin colored by Z-position defined in Fig. 1C. 3X real time. Scale 500nm.

Supplementary Movie 3 Fluorescent movie of MyoVa cargo complex turning at a 2D actin intersection. Cargo complex yellow; actin colored by Z-position defined in Fig. 1C. 4X real time. Scale 500nm.

Supplementary Movie 4 Fluorescent movie of MyoVa cargo complex continuing straight at a 2D actin intersection. Cargo complex yellow; actin colored by Z-position defined in Fig. 1C. 4X real time. Scale 500nm.

Supplementary Movie 5 Model simulation of a cargo complex with a diameter of 350nm and 10 total myoVa motors continuing straight after encountering a 3D actin filament intersection with a filament separation of 100nm. Of the 10 total myoVa motors on each cargo complex, only those which are bound to actin are visualized. Further model description explained in supplementary text. Originally bound filament green; intersecting filament red; myoVa yellow; lipid cargo blue.

Supplementary Movie 6 Model simulation of a cargo complex with a diameter of 350nm and 10 total myoVa motors turning after encountering a 3D actin filament intersection with a filament separation of 100nm. Of the 10 total myoVa motors on each cargo complex, only those which are bound to actin are visualized. Further model description explained in supplementary text. Originally bound filament green; intersecting filament red; myoVa yellow; lipid cargo blue.
