# Supplementary material for: Myosin Va molecular motors manoeuvre liposome cargo through suspended actin filament intersections in vitro
Source: Nat Commun. 2017 Jun 1;8:15692. doi: 10.1038/ncomms15692 (PMC5461480; doi:10.1038/ncomms15692)
Supplement: Supplementary Information — Supplementary Figures, Supplementary Table, Supplementary Methods and Supplementary References [file ncomms15692-s1.pdf]

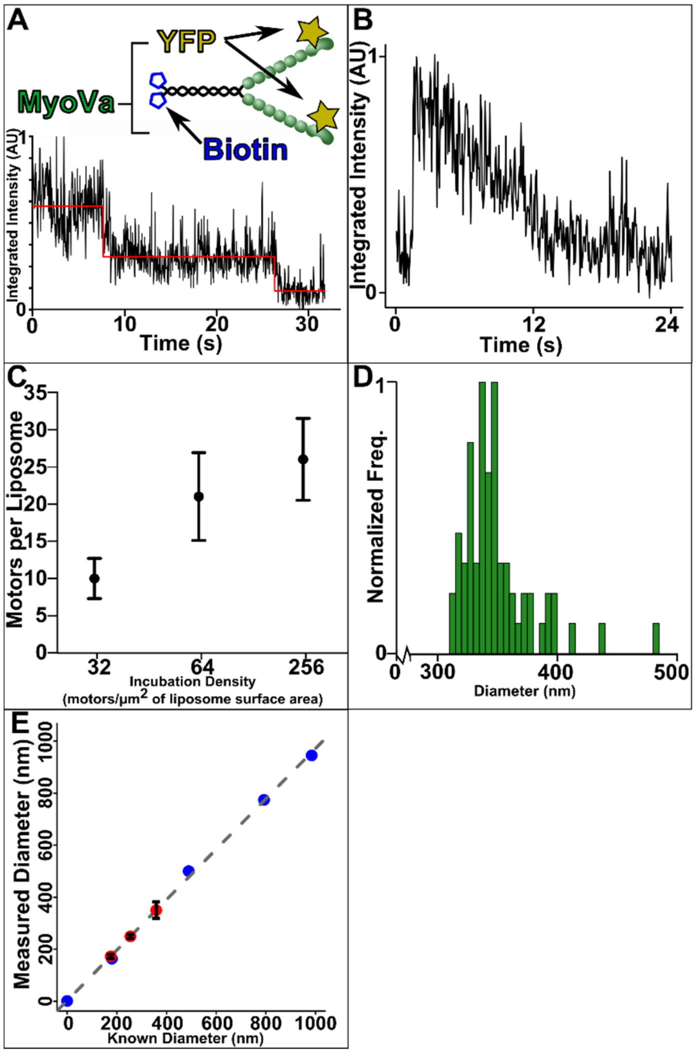


**Supplementary Figure 1.**

Determination of Liposome Size and Motor Density. (A) Cartoon of the expressed myosin Va HMM construct with a C-terminal biotin and a N-terminal YPF. Representative fluorescence photobleaching time course for a single myoVa. Two distinct stepwise photo-bleaching events indicate the presence of two YFP per myoVa dimer. (B) Time course of integrated YFP fluorescence intensity decay for a 350nm liposome coupled to multiple YFP-labeled myoVa at an incubation density of 32 motors/μm$^2$ of liposome surface area. (C) Results for number of myoVa motors per liposome assuming two YFPs per myoVa dimer (mean±s.d.). (D) Normalized frequency histogram of measured liposome diameters by Dynamic Light Scattering (DLS) for liposomes assumed to be 350nm diameter. (E) Graph of DLS measured liposome diameters (red circles) made with three different sized extrusion filters compared to DLS measured diameters of

National Institute of Standards and Technology (NIST) traceable size standard polystyrene beads (blue circles) The standards were used to create a calibration fit line (Dashed). Based on the calibration, liposomes extruded through 200nm, 400nm and 650nm filters gave the following estimated liposome diameters: 172.4 ±7.6nm, 249.2 ±7.9nm, and 350.1 ±31.9nm (mean ±s.d.).

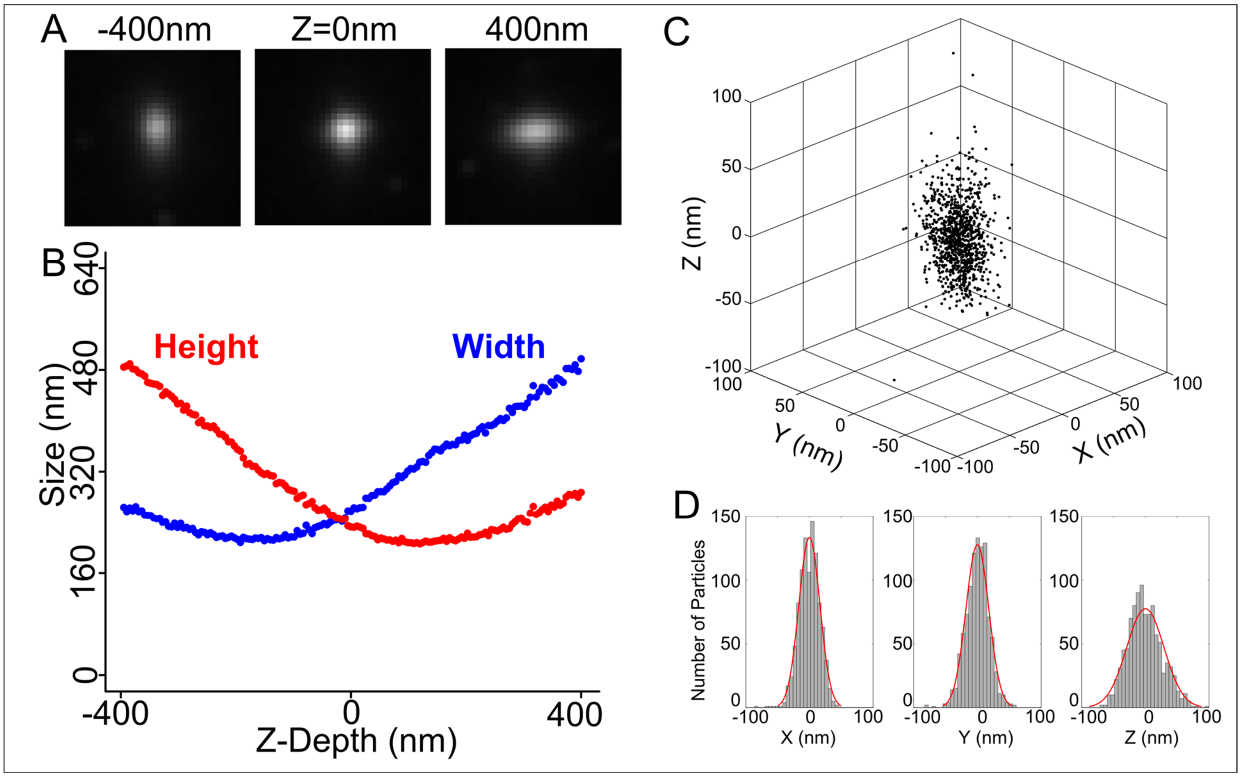


**Supplementary Figure 2.**

Z-position determination. (A) Fluorescence images showing the change in ellipticity with Z-position as a result of introducing a cylindrical lens into the light path. (B) Calibration curve of image heights and widths as a function of Z for 350nm liposomes bound to actin filaments by myoVa motors in rigor (0nM ATP). Each point represents the average from 4 liposomes. (C) Three-dimensional localization of a fluorescent stationary liposome bound to suspended actin. (D) Histograms of the distribution in X ,Y, and Z and Gaussian fits to the distribution overlaid. The standard deviation of the fits are 17nm in X, 18nm in Y and 30nm in Z.

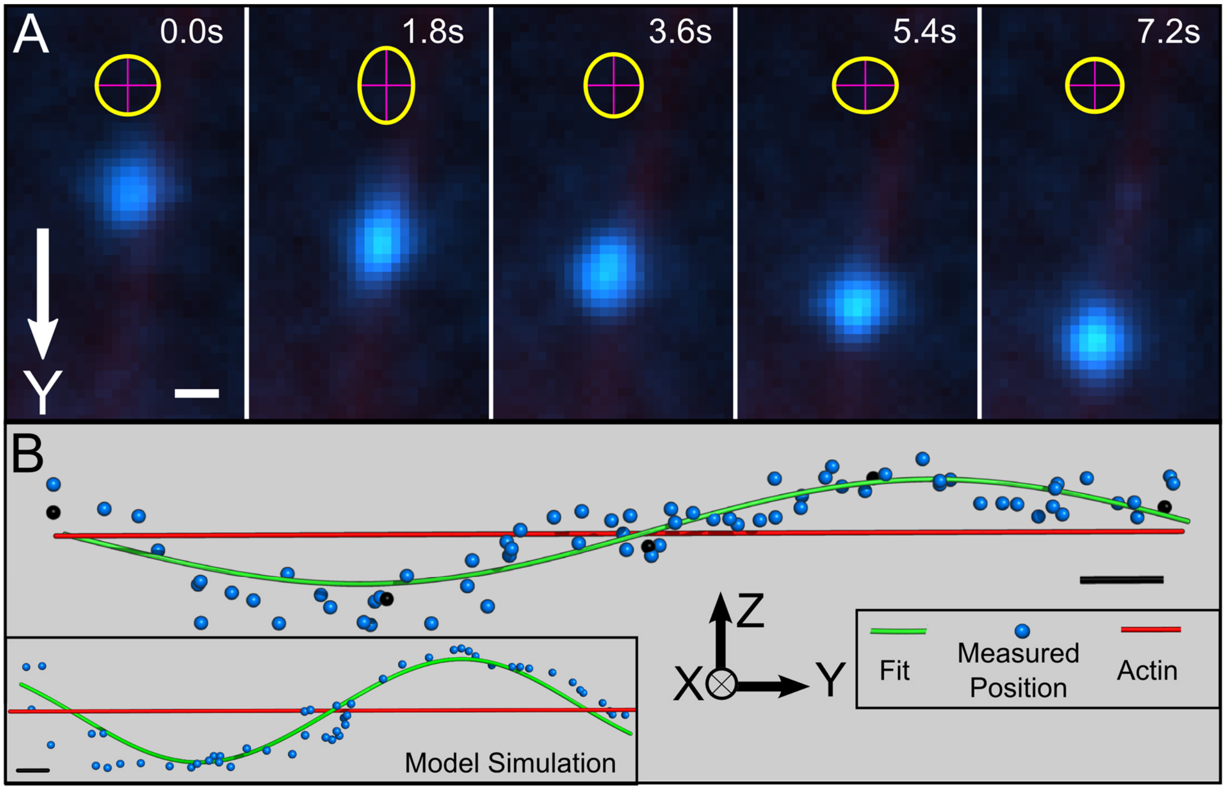


**Supplementary Figure 3.**

3D tracking of motor-cargo complexes on suspended actin filaments. (A) Time sequence of a fluorescently-labeled liposome (blue) image with optically induced astigmatism allowing the Z-position to be defined as the liposome moves along an actin filament. Yellow circle with crosshairs shows the elliptical fit to the liposome's astigmatic image. Scale: 500nm (B) XYZ plot of the entire spiral trajectory in A with black points corresponding to specific images in A. The inset shows that the model simulates the left-handed spiral trajectory observed experimentally. Scale: 100nm.

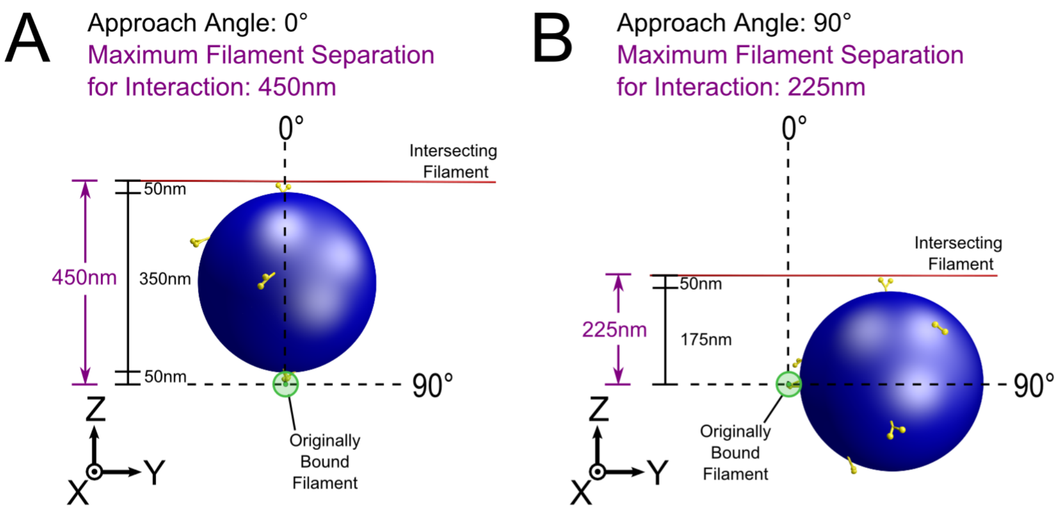


**Supplementary Figure 4.**

Illustrations showing examples of spatial geometries where the filament separation leading to a motor-cargo complex interacting with the intersecting filament is dependent on the approach angle. (A) At approach angle of 0$^{o}$, the cargo is on the same side of the originally bound filament as the intersecting filament. The maximum filament separation at which a surface bound myosin could still reach the intersecting filament is a sum of the 50 nm length of a myoVa HMM [1] bound to the original filament, the 350nm diameter of the liposome and the 50 nm length of a free motor on the surface of the liposome for a total of 450nm. Filament separations above 450 nm at this approach angle would not result in motor-cargo complex interacting with the intersecting filament. When calculated across all approach angles the corresponding filament separation that is predicted to result in an interaction defines the line in the polar plots (Figs. 2B, 3) that is the boundary between a predicted interaction or not. (B) As approach angle increases, the cargo rotates around the originally bound filament. At an approach angle of 90$^{o}$, the 50 nm length of the myoVa attached to the originally bound filament is purely in the Y-Direction and thus does not add to the filament separation predicting a motor-cargo complex interaction with the intersecting filament at this approach angle. Instead, the maximum filament separation for an interaction is the sum of the 175nm radius of the liposome plus the 50 nm length of a free motor on the surface of the liposome for a total of 225nm. Because the line demarcating an interaction or not in the polar plots (Figs. 2B, 3) depends on approach angle, which is an effect of rotating around the originally bound filament, this boundary line calculation is sinusoidal in nature (equation (0)).

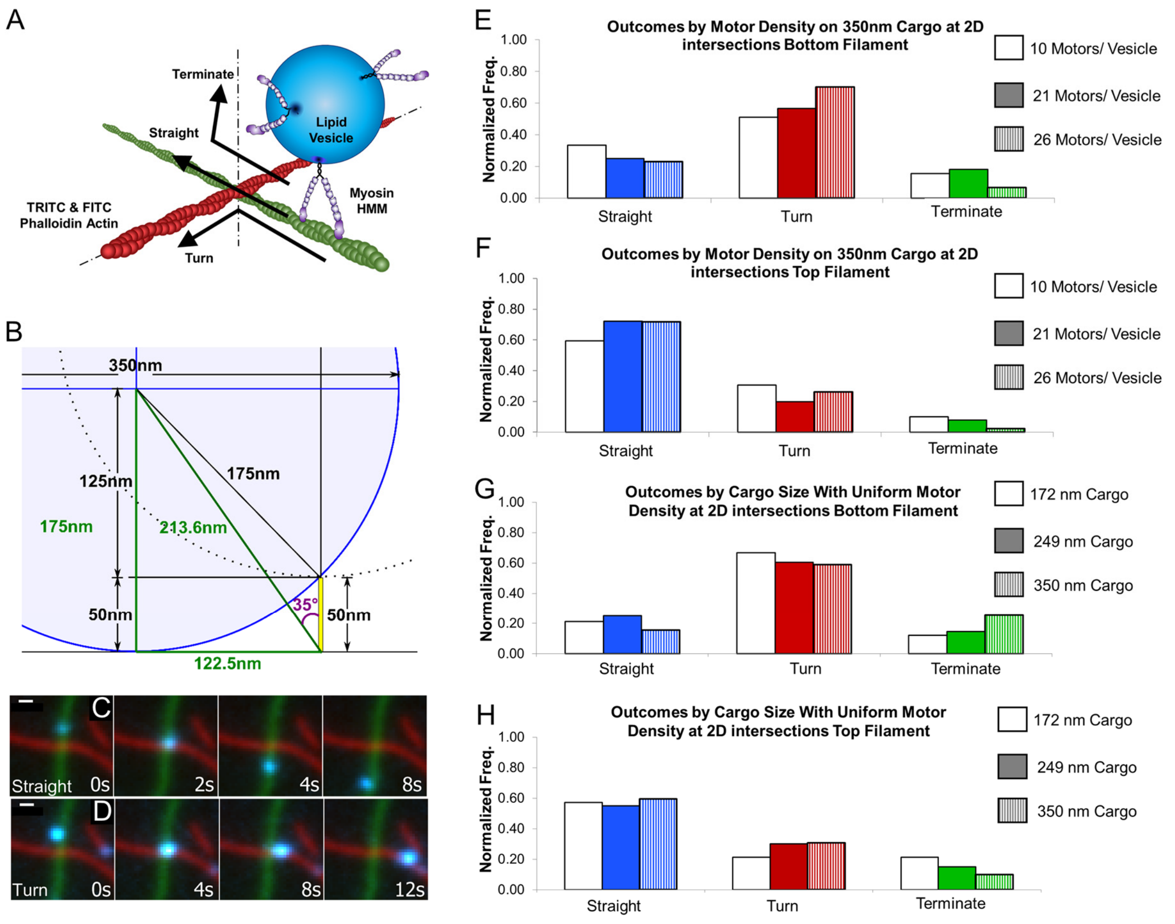


**Supplementary Figure 5.**

Surface bound, two-dimensional, actin filament intersections navigated by myoVa-liposomes of varying size and motor density. (A) Illustration of set up and possible directional outcomes. Both actin filaments are tightly bound to the glass surface with known sequence of filament addition to the flow cell placing the red, TRITC-labeled filament on top of the green, FITC-labeled filament. (B) To scale cartoon indicating the maximum approach angle of ±35° possible at a 2D intersection due to the glass surface. (C) Representative time course images of a straight and (D) a turn directional outcome. Lower actin filament (green); upper actin filament (red), myoVa bound lipid cargo (blue). (E) Directional outcomes at varying surface motor densities on constant sized liposomes from liposomes approaching only from the bottom filament and (F) only from the top filament. (G) Outcomes at varying lipid cargo sizes with constant incubation motor density of 32 motors/μm$^2$ of liposome surface area (Supplementary Figure 1C) from liposomes approaching only from the bottom filament and (H) exclusively on the top filament. Scale 500nm.

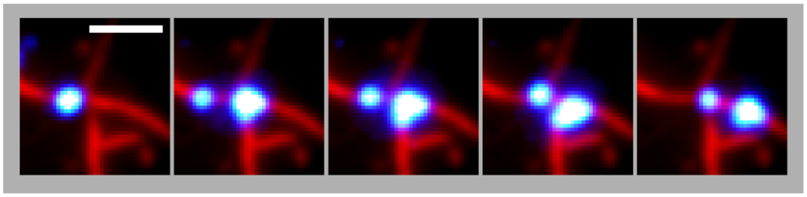

**Supplementary Figure 6.**

Time sequence from left to right of liposome at a 2D actin intersection under conditions promoting very large (>350nm) and high motor density (>20 motors) liposomes (Supplementary Figure 1). The liposome (blue) begins to split between the two actin filaments (red) until the tug of war is resolved and the vesicle snaps to the upper filament. Scale 2000nm.

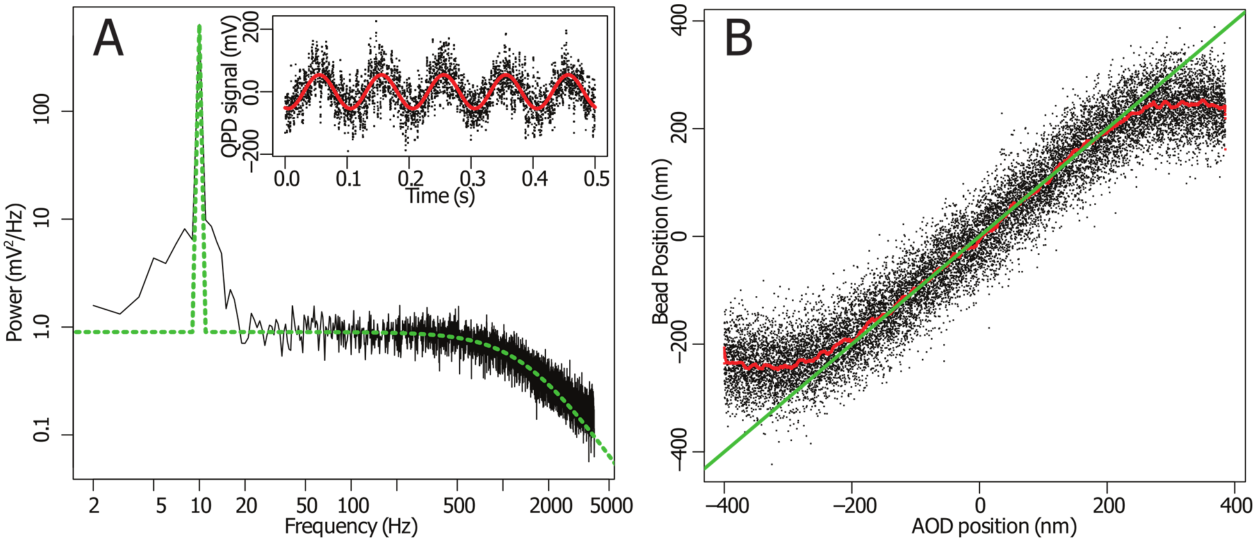


**Supplementary Figure 7.**

Laser trap calibration and characterization of detector response. (A)Representative power spectrum for measurement of trap stiffness and detector response calibration. Dashed green line indicates fit of Eq. 9 from Tolic-Norrelykke et al. (2006)[2]. For every trapped bead, the AOD is utilized to oscillate trap position in a sinusoid with a frequency of 10Hz and an amplitude of 113.6nm (inset). Resulting spike at 10Hz in power spectrum indicates detector response of 210.7nm/V for this particular bead. Coupled with the frequency "rolloff" (1385Hz in this example) indicates a trap stiffness of 0.0235pN/nm. (B) Characterization of linear range of QPD detector. Using the AOD, a trapped lipid-coated bead was scanned across the full range of the detector. The resulting signal (black) was smoothed with a running median filter (100ms wide window, red line). The 1:1 line (green) shows that the detector response is linear (to within 30nm) over a range of 501nm (-234nm to 267nm on this coordinate system).

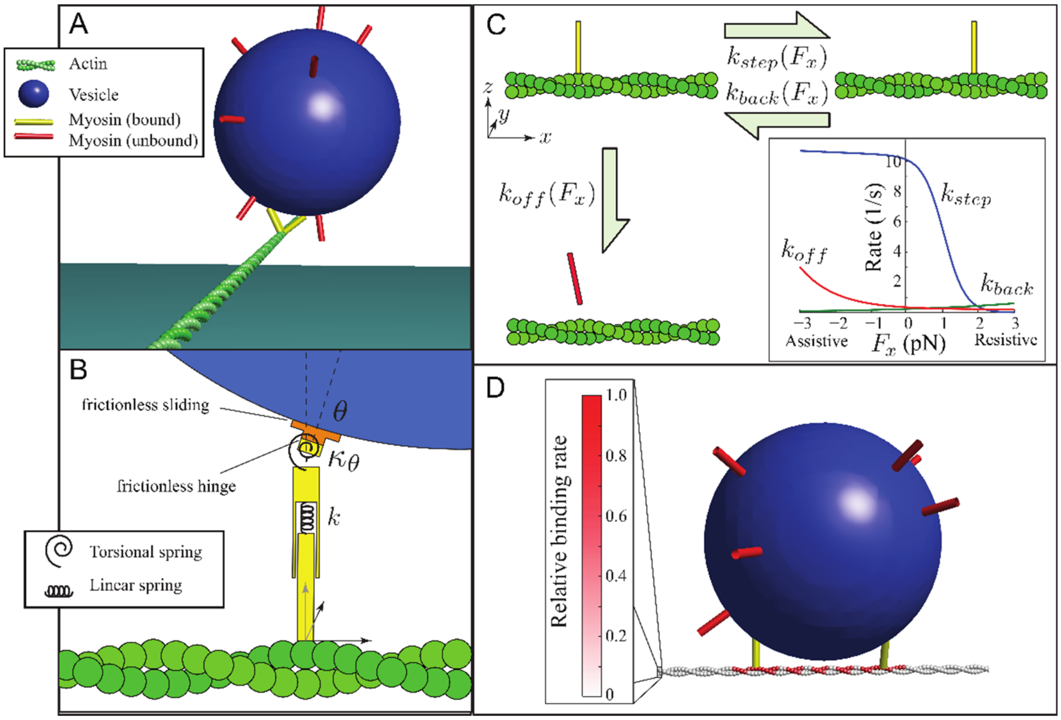


**Supplementary Figure 8.**

Model of an ensemble of myosin Va motors transporting a fluid vesicle along an actin filament. A. The mechanical model. Myosin motors, when not bound to actin (red), diffuse over the surface of the rigid vesicle (blue), whose surface is fluid. The motors can bind to the rigid actin filament (bound motors in yellow, actin in green). These bound motors then step along the actin filament. As they step, the motors experience forces, defined by a simple mechanical model. B. Simple mechanical model of a myosin Va motor. Each motor contains a linear and a torsional spring, that resist extension and bending, respectively. C. Mechanochemistry of myosin stepping and detachment. Each motor, once bound, can step forward, step backward or detach. These rates depend on the amount of force, directed along the actin filament, that is applied to the motor. D. Mechanochemistry of myosin attachment. Myosin's attachment to a specific actin binding site depends on the mechanical energy it would take for a myosin to bind, with a higher energy cost making binding less likely. In this sample calculation, two myosin molecules are bound to actin and the relative attachment rate of a third motor at any actin binding site is color coded according to the color bar. The attachment rate is scaled so that the maximum attachment rate is 1.

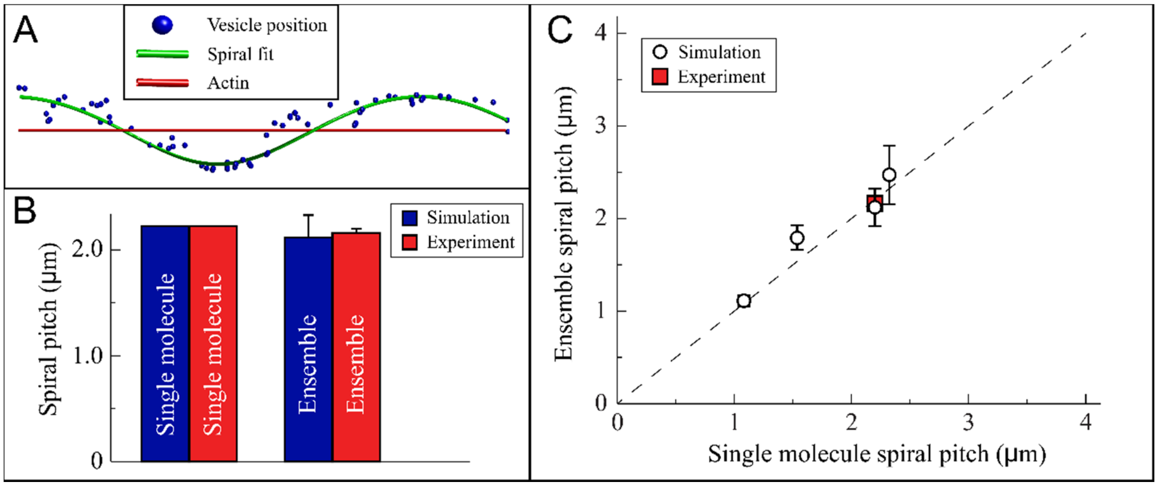


**Supplementary Figure 9.**

Myosin ensembles transport cargo along a left-handed spiral, because each myosin motor occasionally takes a short step. A. An example spiral trajectory. The position of the center of the vesicle (blue sphere) is shown every 0.1 second. A left-handed spiral, with a pitch of 2.29 µm (green), reasonably describes this trajectory. B. Vesicles transported by a myosin ensemble follow spiral trajectories with the same pitch as vesicles transported by a single myosin motor. The single molecule simulation result is calculated from the probability of myosin motors stepping one actin monomer short of the actin periodicity 22% of the time (ps = 0.22). C. Experimental results are from Ali et al.[3] (single molecule) and from our experiments (ensemble). The probability of myosin motors stepping one actin monomer short of the actin periodicity predicts both single molecule and ensemble spiral pitch. We performed four sets of simulations, varying the probability of myosin motors stepping one actin monomer short of the actin periodicity (ps = 0.21, 0.22, 0.32 and 0.45). In all simulations, ensemble spiral pitch was similar to calculated single molecule spiral pitch (dashed line). Ensemble simulation results are from ten simulations of 300 chemical reactions, mean distance traveled divided by mean number of rotations. Error bars show standard error, propagated from standard error in mean distance and mean number of rotations.

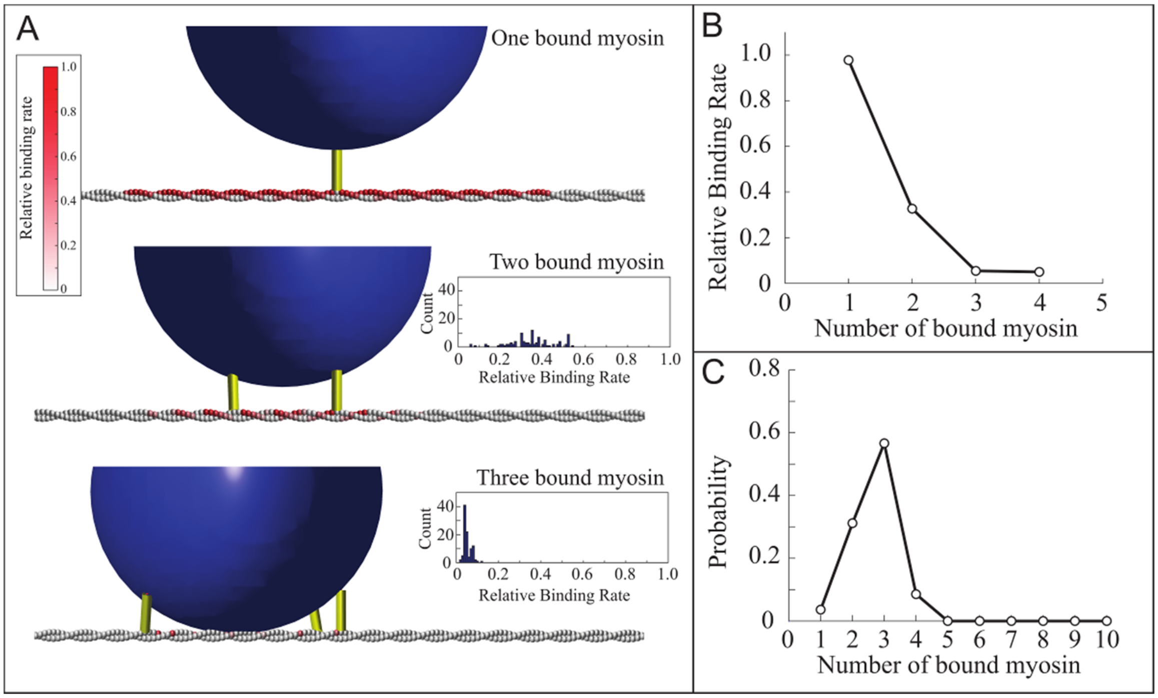


**Supplementary Figure 10.**

Negative cooperativity in myosin binding to actin. A. Calculation of attachment rate to binding sites on actin (Eq. 6) with one (top), two (middle) and three (bottom) myosin motors bound shows that the binding of myosin motors restricts the access of subsequent motors to actin binding sites. When two or three myosin motors are bound to actin, attachment depends on the relative position of the motors. Insets show a histogram of the overall attachment rate (the sum of the attachment rate over every actin binding site), relative to the overall attachment rate of a second motor, $k_a$. One hundred motor configurations are shown in each histogram. B. The average attachment rate of an unbound myosin molecule strongly decreases as the number of actin bound motors increases. The average overall attachment rate when 1-4 myosin motors were bound to actin was calculated for five simulations of motor ensembles transporting a fluid vesicle. The overall attachment rate, averaged over all observed motor configurations, is shown relative to the overall attachment rate of a second motor $k_a$. C. Strong negative cooperativity makes it rare for more than three motors to be simultaneously bound to actin. For five simulations, we determined the proportion of time that 1-10 myosin motors were simultaneously bound to actin. Simulation parameters are given in Supplementary Table 1.

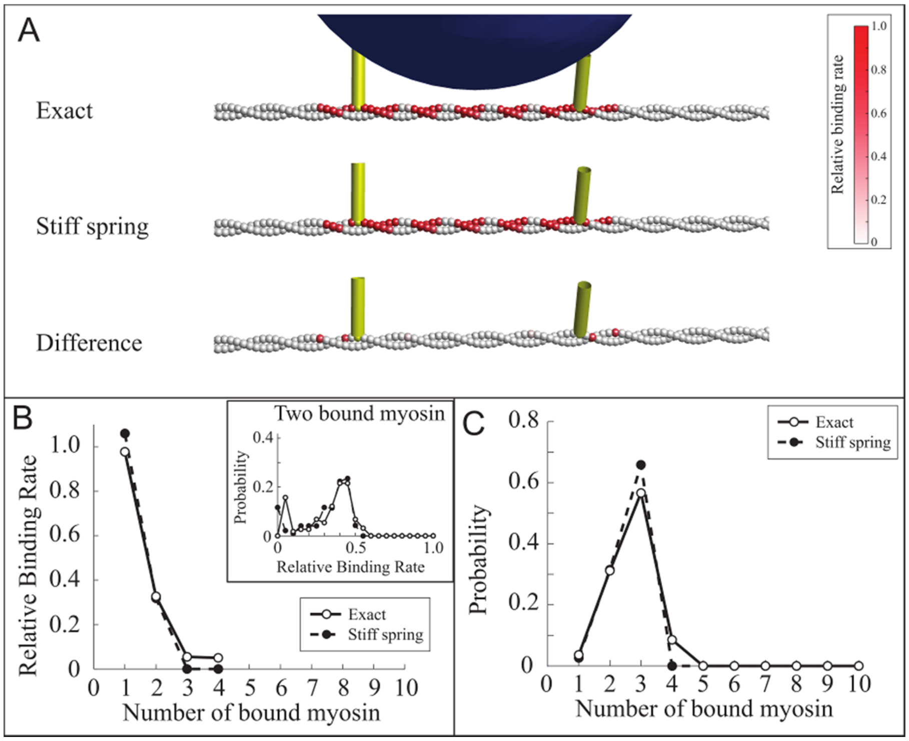


**Supplementary Figure 11.**

A stiff spring approximation reasonably describes the full model and greatly increases computational efficiency. A. The stiff spring approximation describes the attachment rate to each binding site along actin. For two bound myosin molecules, an exact calculation of the binding rate of a third myosin molecule to each actin binding site (top) compares well with a calculation that assumes myosin is inextensible (middle) and the difference between the two is small (bottom). B. The stiff spring approximation captures the negative cooperativity of myosin motors binding to actin. Average overall attachment rate when 1-4 myosin motors were bound to actin was calculated for five simulations of motor ensembles transporting a fluid vesicle both using the exact model (solid line, hollow symbols) and using the stiff spring approximation (dashed line, solid symbols). The attachment rate is averaged over all observed motor configurations and shown relative to the overall attachment rate of a second motor calculated using the exact calculation ($k_a$). Inset shows a histogram of overall attachment rates when two myosin motors are bound to actin, demonstrating that the stiff spring approximation captures the distribution of attachment rates along with the average. C. The stiff spring approximation describes the proportion of time that different numbers of myosin motors are bound to actin. For five simulations, we determined the proportion of time that 1-10 myosin motors were simultaneously bound to actin using both the full

model (solid line, hollow symbols) and the stiff spring approximation (dashed line, solid symbols). Agreement is reasonable, and the simulations take minutes rather than days. Simulation parameters are given in Supplementary Table 1.

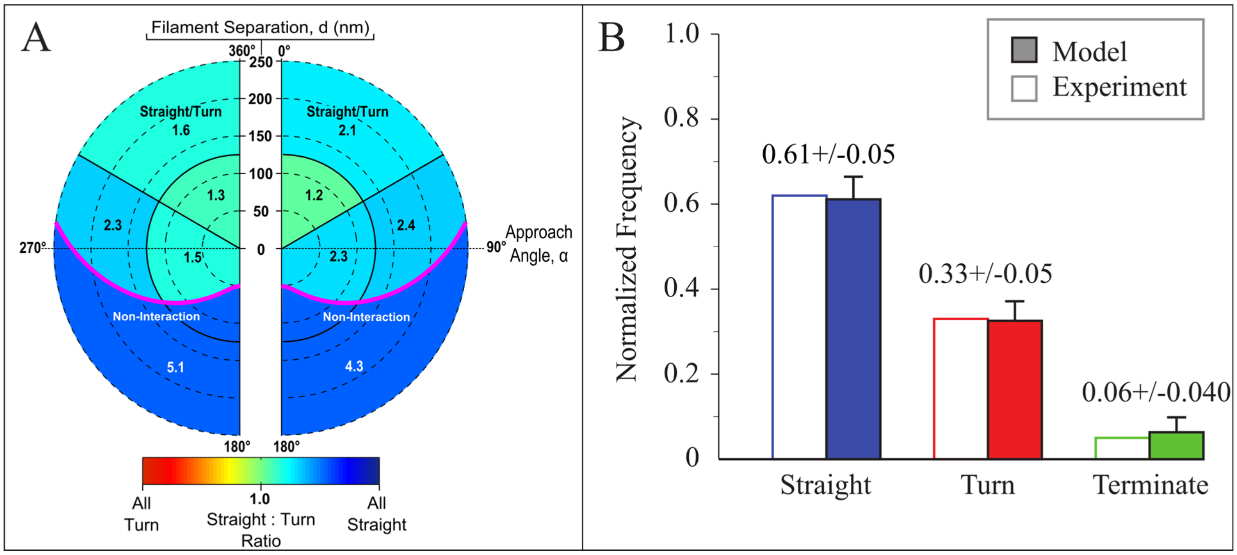


**Supplementary Figure 12.**

The model captures outcomes observed when myosin motor teams transporting a fluid vesicle encounter an actin intersection in 3D. A. Results of a single simulated data set, showing approach angle from 0 to 360 degrees and filament separation. Results between 0-180 degrees and 180-360 degrees are nearly identical. As in the experiments, there is no obvious relationship between filament separations, approach angle and whether the vesicle terminates, turns or goes straight; however, if the motors cannot interact with the crossing filament, they go straight. B. The accumulated results of ten simulation runs agree with our experimental measurements. Of the outcomes where an interaction between the motors and the crossing filament was predicted, the simulations show a preference for proceeding straight through the intersection, in agreement with our experiments. Error bars are standard deviation; simulation parameters are given in Supplementary Table 1.

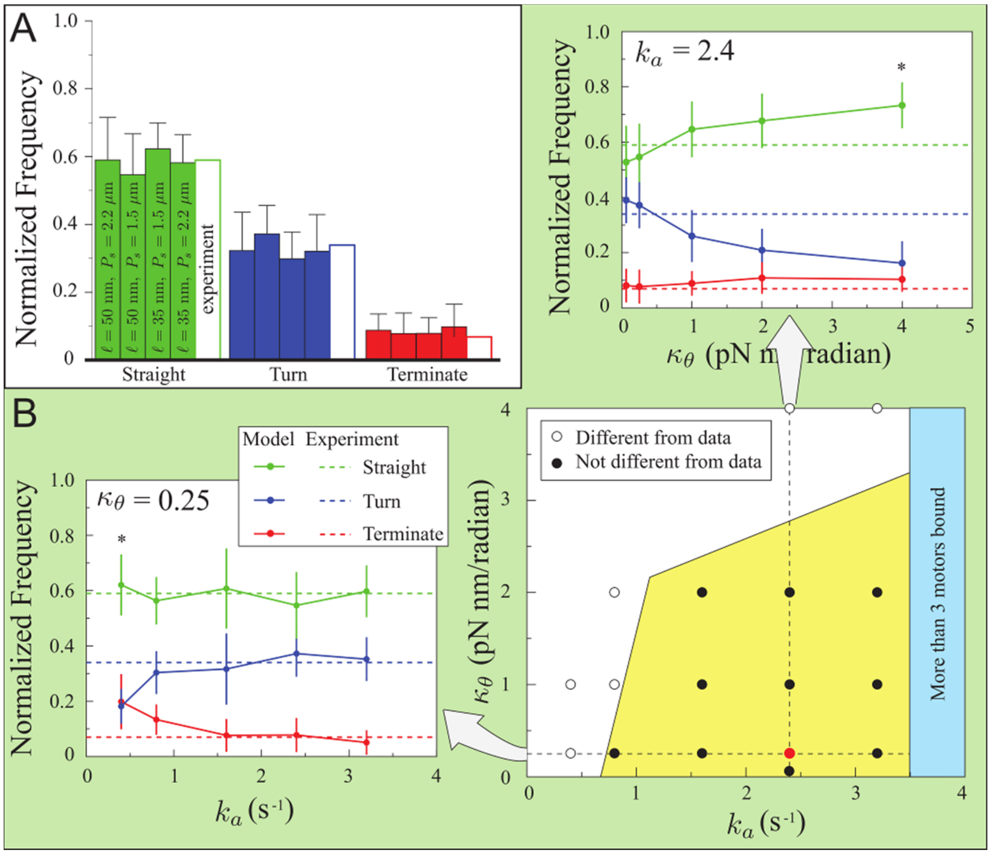


**Supplementary Figure 13.**

Simulated fluid vesicles transported by a myosin ensemble prefer to go straight through a 3D actin intersection for a wide range of model parameters. A. Varying myosin's rest length, $\ell$, and spiral pitch, $P_S$, has little effect on directional outcomes. Simulations with a shorter myosin rest length $\ell$ = 35 nm and/or more frequent short steps $p_s$ = 0.33 (giving a spiral pitch, $P_s$ = 1.5 µm) are not significantly different from measurements ($p$>0.05, $\chi^2$ test). B. Varying myosin's attachment rate, $k_a$, and torsional stiffness, $\kappa_\theta$, affects directional outcomes but the majority of outcomes are straight. Bottom right shows the results of a series of simulations where $k_a$ and $\kappa_\theta$ were varied systematically. Each symbol represents ten simulations of experimental measurements, with filled symbols showing simulations that are consistent with measurements ($p$>0.05, $\chi^2$ test) and hollow symbols showing simulations that are not consistent with measurements ($p$<0.05, $\chi^2$ test). The red symbol shows the parameters used in our simulations. These simulations identify a region of parameter space that is consistent with our measurements (yellow). As attachment rate gets too large, more than 3 myosin motors will bind (blue). Plots at top and left show directional outcomes

with variable $k_a$ and $\kappa_\theta$ fixed at 0.25 pN nm rad$^{-1}$ (left) and variable $\kappa_\theta$ and $k_a$ fixed at 2.4 s$^{-1}$ (top). All simulations show a majority of straight trajectories. Parameters not specifically mentioned are as listed in Supplementary Table 1, except $p_s$ = 0.33 (giving a spiral pitch of $P_s$ = 1.5 μm). Outcomes reported are mean plus/minus standard deviation for 10 simulations. *Significantly different from experimental measurement, $p$<0.05, $\chi^2$ test.

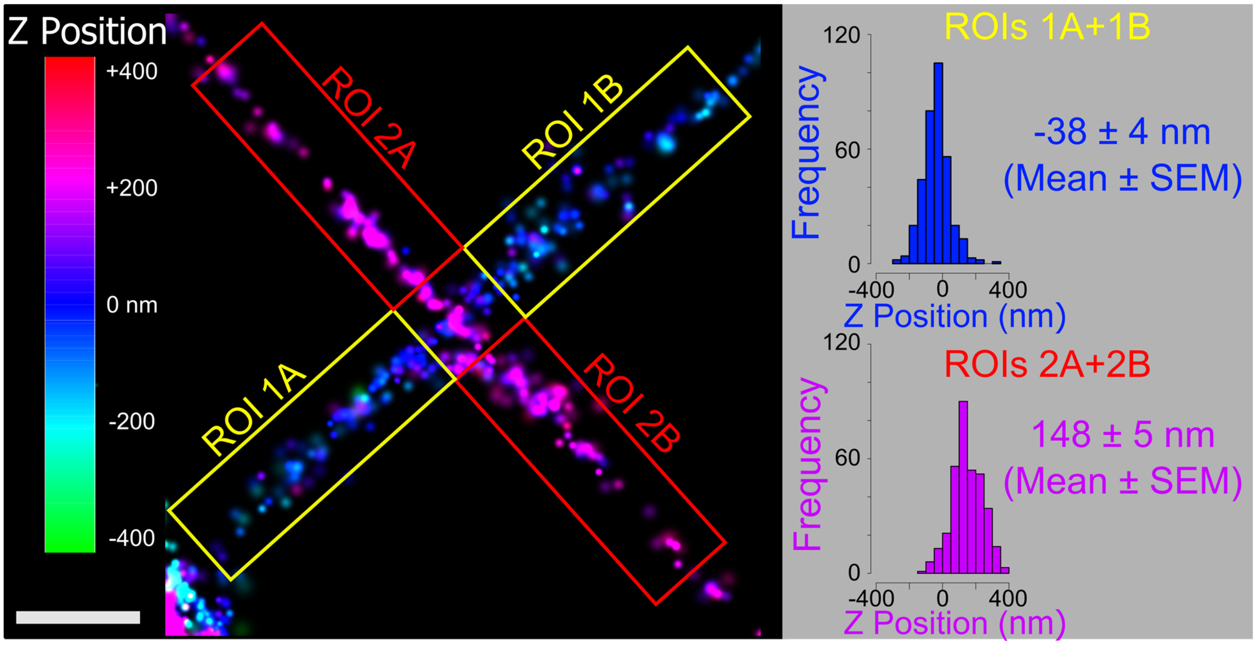


**Supplementary Figure 14.**

Determination of actin filament separation at an intersection. Left: A 3D STORM image reconstruction from Figure 1E showing the suspended intersecting actin filaments at different Z-positions, as indicated by color (filament 1, blue; filament 2, magenta). Four regions of interest (ROIs) define spatial volumes on all 4 sides of the intersection. Right: Histograms of Alexa 647-phalloidin Z-positions associated with each filament's combined ROIs (i.e., filament 1 (ROI 1A, 1B), filament 2 (ROI 2A, 2B)) are shown. These histograms suggest that the Alexa 647 fluors are uniformly distributed in Z-position about the filament center. Therefore, each filament's Z-position was taken as the mean Z-position for all fluors within the histograms and that the standard error of the mean (s.e.m.) estimated the precision. For this example, the filament separation was 186 ± 6.4 nm with the error determined by propagating the error associated with each filament's Z-position (see above). Scale 500nm.

**Supplementary Table 1: Model Parameters**

| Symbol | Value | Description | Justification |
|---|---|---|---|
| $R$ | 175 nm | radius of vesicle | measured |
| $N_M$ | 10 | number of myosin motors/vesicle | measured |
| $k$ | 1 pN/nm | myosin's stiffness in extension | estimated from Vilfan (2005)[4] |
| $k_B T$ | 4.14 pN nm | Boltzmann's constant times temperature | calculated |
| $\mu$ | $\rightarrow\infty$ | vesicle's fluidity | assumed ideally fluid |
| $\gamma$ | $3.3 \bullet 10^{-6}$ pN s/nm | viscous drag constant for vesicle | Stokes' drag |
| $s$ | 1.25 | scaling factor for myosin's reactions | fits to measured velocity |
| $p_s$ | 0.22 | probability of myosin taking a short step | gives spiral pitch of 2.2 μm |
| $\ell$ | 50 nm | rest length of myosin | estimated from Vilfan (2005)[4] |
| $\kappa_\theta$ | 0.25 pN nm/rad | stiffness of myosin's torsional spring | from fits/sensitivity analysis |
| $k_a$ | 2.4 $s^{-1}$ | attachment rate of second myosin | from fits/sensitivity analysis |

**Supplementary Methods**

1. A model of 3D transport by multiple myosin Va motors introduction

To better understand and interpret our measurements of vesicles being transported by myosin Va motor teams, we constructed a mathematical model of the physical system. While the model makes some simplifying assumptions, our aims were to both capture the essence of the system and to make the model physically consistent. The following is a discussion of model assumptions and additional details of model results to supplement the main text.

2. Model and model assumptions

The model contains three components, the vesicle, the actin filament(s), and the myosin Va motors. Each myosin motor undergoes chemical reactions (i.e. stepping forward and backward along actin and detaching from actin) whose rate depends on the force applied to the motor. Thus, to define the model, we must determine the forces on the motors as they attach to, detach from and step along the actin filament(s). To determine these forces, we must create a mechanical model of each of the three components.

*Component 1: Mechanical model of the vesicle*

We model the vesicle as a rigid sphere, of radius $R$ (Supplementary Figure 8A). The surface of the vesicle is fluid, so that forces tangent to its surface cause the membrane to flow. Contact between the vesicle and the actin filaments is frictionless. There is no attraction or repulsion between the vesicle and the actin filaments, other than steric constraints that keep the two from occupying the same space.

*Component 2: Mechanical model of myosin*

We model each myosin Va molecule as an extensible rod, of rest length $\ell$, anchored into the fluid surface of the vesicle by a deformable pivot (Supplementary Figure 8B). The motor's extensibility is linear, with spring constant k. The pivot, that connects the myosin motor to the vesicle, contains a universal joint with a torsional spring of stiffness $\kappa_\theta$ (Supplementary Table 1). There is also a frictionless hinge that allows the motor to freely rotate about an axis normal to the surface of the vesicle. The combination of pivot and torsional spring allows us to model the bending stiffness of the myosin molecule. In particular, the spring and pivot work together to cause the motors to extend in the normal direction off of the vesicle and resist angular deflections in any direction equally.

Naturally, myosin Va is not really an extensible rod. Thus, myosin's rest length, $\ell$, and spring constant, k, might vary depending, say, on whether one or two heads are bound to actin, or on the state of each head. Vilfan estimates ~ 34 nm for the total length of each lever arm and myosin head[4]. Since the coiled-coil might add a little to this length, we estimate $\ell$ = 50 nm (we examine this assumption further in section 3.4). Vilfan [4] also estimates the stiffness of a single lever arm to be around 0.25 pN/nm when force is applied perpendicular to its long axis. When both heads are bound, and force is applied vertically, stiffness should be higher than twice this value, so we estimate k = 1 pN/nm.

When attached to actin, the motors can experience forces. Since the vesicle is fluid, tangential components of these forces, $F_T$, cause the motors to move relative to the vesicle surface at a velocity $v = \mu F_T$, where μ is a drag coefficient. The anchoring point of the motor diffuses across the surface of the vesicle with diffusion constant $D = \mu k_B T$, where $k_B$ is Boltzmann's constant and $T$ is the absolute temperature[5].

As we are interested in how motor teams transport fluid vesicles, we make the simplifying assumption that the vesicles are "ideally fluid." That is, $\mu \rightarrow\infty$, so that diffusion is very fast and motors experiencing tangential forces slide very quickly. This assumption contrasts with an "ideally solid" vesicle, where motors are anchored rigidly to the surface ($\mu \rightarrow 0$). The measured value of the diffusion constant, $D$ = 0.92 μm$^2$/s *(ref. 5)* giving $\mu$ = 2.22x10$^5$ nm/pN·s, shows that diffusion is fast and that even modest tangential forces (~1pN) cause rapid sliding velocities (~ 200 μm/s).

*Component 3: Mechanical model of actin*

We model each actin filament as a rigid rod that is held rigidly in place. Myosin binding sites are arrayed every 5.5 nm along a right-handed double helix, with a periodicity of 72 nm (~ 14 monomers). Each binding site has a specific orientation, so that a myosin motor, once bound, extends rigidly from the binding site. This preferred orientation, which is orthogonal to the actin filament, rotates azimuthally with the actin periodicity (one full rotation every 72 nm, or 14 monomers).

*Calculating forces: rapid mechanical equilibrium*

Generally, the equations of motion for a small sphere in water are given by the Langevin equation

$$0 = -\gamma \frac{dx}{dt} + F_{ext} + F_f \qquad (1)$$

where the first term on the right hand side is the viscous force on the vesicle, with $\gamma$ being a drag constant, $\boldsymbol{F}_{ext}$ is the sum of the external forces on the vesicle and $\boldsymbol{F}_f$ is a stochastic fluctuating force, due to solvent collisions. Since the fluctuating force is stochastic, this is a stochastic ordinary differential equation (ODE) and can be re-written as a partial differential equation (PDE) in terms of a probability density ($\rho(\boldsymbol{x},t)$), the Smoluchowski equation

$$\frac{\partial \rho}{\partial t} = \nabla \cdot \left( \frac{k_B T}{\gamma} \nabla \rho + \frac{\rho}{\gamma} \nabla V \right) \qquad (2)$$

where $V(\boldsymbol{x})$ is the potential giving rise to $\boldsymbol{F}_{ext}$, such that $-\nabla V = \boldsymbol{F}_{ext}$.

In steady-state, the solution to this equation is $\rho = N_C \exp(-V/k_B T)$, where $N_C$ is a normalization constant. This steady-state probability distribution is a good approximation of $\rho$ for times that are long compared to the characteristic time scale $\gamma L^2/k_B T$, where $L$ is a length scale, defined by the potential $V$. When one or several motors move the vesicle, we expect that it will typically move $L \approx 36$ nm when a motor takes a step, or $L < R$ when a new motor attaches. Thus, the upper limit of this time scale is $\gamma R^2/k_B T \approx 0.024$ s (assuming Stokes' drag on the vesicle, and a

vesicle radius $R = 175$ nm), and a more typical value is $\gamma(36\text{ nm})^2/k_BT \approx 0.0010$ s. Typical vesicle velocities in our experiments were $v \approx 450$ nm/s, giving a stepping time scale of 0.08 s, 3-80 times longer than these time scales. Therefore, we expect the steady-state solution $\rho \approx N_C \exp(-V/k_BT)$ to be a good approximation.

Thus, in the model, we assume that $\rho = N_C \exp(-V/k_BT)$. To do so, after each chemical reaction (i.e. a myosin step, attachment or detachment), we calculate the new position of the vesicle/motor system by finding mechanical equilibrium (the position that minimizes $V$). Since there may be times where multiple minima of $V$ exist, we ensure that we find the most likely minimum by simulating the Langevin equation (Eq. 1) neglecting the fluctuating force $\boldsymbol{F_f}$, until the system approaches mechanical equilibrium. We then perform a numerical root-find to solve $\boldsymbol{F_{ext}} = \mathbf{0}$. These resulting forces allow us to calculate the forces on each attached myosin motor.

*Mechanochemical model of myosin*

In the model, myosin motors can perform forward steps at a rate $k_{step}$, backward steps at a rate $k_{back}$ and detach at a rate $k_{off}$. All of these rates depend on the component of force on the myosin molecule that points along the axis of the actin filament, $F_x$ (Supplementary Figure 8C). This force is assumed positive if in the direction opposing forward steps (e.g. Nelson et al.[5]). The reaction rates are (in $s^{-1}$)

$$k_{step} = \frac{1}{s}\left(\frac{exp\left[{}^{0.04F_x}/_{k_BT}\right]}{13} + \frac{exp\left[{}^{14F_x}/_{k_BT}\right]}{426}\right)^{-1} \qquad (3)$$

$$k_{back} = \frac{1}{s} 0.3exp\left[{}^{1.3F_x}/_{k_BT}\right] \quad (4)$$

$$k_{off} = \frac{1}{s}(0.1724exp[-1.005F_x] + 0.2634) \quad (5)$$

All rates come from Kad et al. 2008 [6] ,but we have introduced a scaling factor, *s*, that allows us to adjust motor velocity to be consistent with our measurements without affecting the motors' run length or stall force.

When a motor steps, the position of the myosin molecule is moved forward 36 nm most of the time, but occasional 31 nm steps occur. These infrequent short steps cause a single myosin motor to describe a spiral trajectory. If the probability of taking a short step is $p_s$, the pitch of this spiral, $P_s$, is the number of steps required to make a full rotation ($14/p_s$) times the average size of a step ($36(1-p_s)+31p_s$), so that $P_s = (14/p_s)(36(1-p_s)+31p_s)$ nm.

Besides stepping and detaching, myosin molecules attach to actin. Given the assumption that diffusion across the surface of the vesicle is fast, this attachment rate for each motor is inversely proportional to the surface area of the vesicle, $4\pi R^2$. Since myosin molecules likely attach to actin via a weak-binding intermediate, we assume that the attachment rate depends exponentially on the mechanical energy it takes for myosin to bind, $\Delta E$[7].

$$k_{att} = \frac{k_0}{4\pi R^2} exp\left[-{}^{\Delta E}/_{k_BT}\right] \quad (6)$$

To determine this attachment rate, we calculate $\Delta E$ for every binding site on actin that is not already occupied with a myosin molecule. So, for a particular binding site, this requires finding the mechanical equilibrium configuration that would result from a motor binding to that binding

site, calculating the energy of that configuration, and subtracting the energy of the current configuration from that value. The results of an example calculation are shown in Supplementary Figure 8D.

Given the vesicle radius, Eq. 6 has one free parameter, $k_0$. Rather than specifying $k_0$ directly, it is more convenient to specify an overall attachment rate, which is the sum of $k_{att}$ over every binding site on actin. Generally, this attachment rate depends on the number of myosin motors on the vesicle surface that are attached to actin, and the relative position of those attached motors. However, when a single myosin molecule is bound to actin, the vesicle is always positioned directly over it. Therefore, we define $k_a$ to be the overall attachment rate when one motor is bound (i.e. the attachment rate of a second motor). We specify this value in our simulations (which then defines $k_0$ in Eq. 6).

To simulate a molecule undergoing these interactions, we use the Gillespie algorithm[8]. Briefly, at each time step, we determine the time of every myosin molecule undergoing every possible chemical reaction (stepping forward, stepping backward, attaching and detaching) by picking random numbers from the appropriate distribution. These times are then sorted, and the reaction with the shortest time is implemented and time is advanced by that time step. In the original method[8], the next shortest reaction would be implemented, time advanced, and so forth. However, since each reaction generally changes the forces on each attached motor, and since the reaction rate constants depend on these forces, after each reaction occurs we re-calculate all reaction times. Importantly, this method gives the correct stochastic fluctuations that occur when a small number of molecular motors transport a shared cargo.

*Model parameters*

The model parameters are summarized in Supplementary Table 1. We can estimate or calculate all but two: $\kappa_\theta$ and $k_a$. To estimate these parameters, we performed a sensitivity analysis (section 3.4).

We also examined the sensitivity of the model to $p_s$ and $\ell$ (see section 3.4). Since the simulation results were insensitive to these parameters, we used parameter values that differ from those in Supplementary Table 1 for some simulations. For example, we performed our sensitivity analysis of $\kappa_\theta$ and $k_a$ with $p_s = 0.33$, which gives a spiral pitch of 1.5 μm. This shorter spiral pitch makes it easier to uniformly cover all possible approach angles.

## 3. Results

We performed a series of simulations of this model of myosin motor ensembles transporting a fluid vesicle in three dimensions. Here we focus on three specific results of these simulations. First, because myosin molecules occasionally take a short step, simulated motor ensembles move vesicles along actin in a left-handed spiral. Second, simulated myosin ensembles exhibit strong negative cooperativity when binding to actin; that is, the binding of one motor strongly decreases the binding rate of the next motor. Third, simulated myosin ensembles prefer to proceed straight through 3D actin intersections, and the probability of going straight, turning and terminating agrees

well with our experimental observations. We now discuss these three results, and then examine how the last result depends on parameters.

*3.1 Results I: spiral trajectories*

Because myosin takes an occasional short step, single myosin Va molecules follow a left-handed spiral trajectory with a pitch of 2.2 μm as they move along actin[9]. Given that most steps are 36 nm, and that these occasional short steps are 31 nm, then a pitch of 2.2 μm corresponds to a probability of taking a short step $p_s$ = 0.22. When we simulated myosin motor ensembles transporting a vesicle along an actin filament, we observed spiral trajectories (Supplementary Figure 9A). These spirals had a nearly identical pitch to the single molecule (2.1 ± 0.2 μm, $N$=10 mean± s.e.m.). This result is consistent with our experimental measurements (Supplementary Figure 9B).

Given the similarity between the spiral trajectories of vesicles being transported by single motors and vesicles being transported by motor ensembles, it seems likely that myosin's occasional short step is responsible for both. To test this idea, we performed a series of simulations with variable short stepping probabilities ($p_s$ = 0.21, 0.22, 0.32 and 0.45, corresponding to single molecule spiral pitches of 2.3 μm, 2.2 μm, 1.5 μm and 1.0 μm, respectively). In all cases, the spiral pitch for motor ensembles was similar to the spiral pitch of a single molecule (Supplementary Figure 9C). We therefore conclude that, in the model, myosin's occasional short step is responsible for the spiral trajectories observed for motor ensembles.

*3.2 Results II: negative cooperativity*

The model predicts strong negative cooperativity when myosin motors bind to actin. In particular, when a single myosin motor is bound to actin, the vesicle can rotate around myosin's pivot (the pivot is shown in Supplementary Figure 8B). Although this rotation is resisted by myosin's torsional spring, the second myosin molecule has access to many binding sites on actin. After that second myosin binds to actin, the motion of the vesicle is restricted, so that it can only rotate around a single axis (the line that connects the pivots of the two attached myosin molecules), and far fewer binding sites are available. Binding of a third myosin molecule effectively freezes the configuration of the vesicle, so almost no binding sites are available ( Supplementary Figure 10A).

In order to characterize this negative cooperativity, we quantified how myosin's attachment rate depends on the number of myosin molecules bound to actin. Adding up the binding rates to each actin monomer gives an overall attachment rate. When more than one motor is bound to actin, the distribution of available binding sites depends on exactly how the motors are positioned relative to one another. Thus, there is a distribution of these overall attachment rates (Supplementary Figure 10A, insets). To characterize how overall attachment rate depends on the number of actin-bound myosin motors, we calculated overall attachment rate for a series of five simulations. We then determined when a given number of motors were bound, and found the average attachment rate. We observed that, if the overall attachment rate is $k_a$ when one motor is bound, it decreases to $0.33k_a$ when two motors are bound and further to $0.05k_a$ when three or more motors are bound (Supplementary Figure 10B).

One result of this negative cooperativity is that even though 10 motors are available to bind, and even though these motors can diffuse over the surface of the vesicle, the model predicts that there are typically a maximum of three motors simultaneously bound to actin. In fact, only 9% of the time are there four motors simultaneously bound to actin (Supplementary Figure 10C), while there are 1-3 motors simultaneously bound to actin the remaining 91% of the time. We never saw more than four motors simultaneously bound. Thus, at an actin intersection, we would not see a tug of war between two large groups of myosin motors, but rather between groups of motors comprised of a single motor and of two motors.

### *3.3 Results III: crossing simulations*

#### *3.3.1 Stiff spring approximation, increasing computational efficiency*

To compare the model to measurements of vesicles encountering actin intersections in 3D, we had to increase the efficiency of the simulations. In particular, calculating of the binding energy for the calculation of attachment rates is computationally expensive. It requires calculating the equilibrium position of the system for myosin binding to each available actin binding site. Finding the equilibrium position is not trivial, since even assuming mechanical equilibrium, one must simultaneously solve three non-linear equations (force balance in 3D). Further, since the solution is generally not unique, one must identify the correct mechanical equilibrium point.

To simplify the calculation, we made the approximation that myosin, as a linear spring, is very stiff. Then, extension of myosin is energetically prohibited. One can then easily find the equilibrium position of the vesicle by solving a geometry problem - i.e. if three myosin molecules

are bound, where is the vesicle such that each myosin molecule is not extended? Solving similar geometry problems for two and one bound myosin, we increase the computational efficiency by roughly $10^3$, so that simulations that would take overnight can be completed in minutes. One important consequence of the approximation is that four motors cannot bind concurrently, unlike the more complex simulations, where the binding of four motors is disfavored, but possible.

To ensure that this stiff spring approximation was valid, we performed five simulations with the approximation (~15 minutes of computer time) and five simulations without the approximation (about a week). The approximation does a reasonable job capturing details of attachment and the distribution of attached motors (see Supplementary Figure 11).

*3.3.2 Simulation results*

We performed a series of experiments, observing motor teams navigating actin intersections in 3D (described in the main text and supplementary information). All actin intersections were at approximately right angles; minimum separation between the filaments varied. Since our model does not allow a single motor to step from one filament to the other, when comparing the model to these experimental data, we only considered experiments where the separation was 50 nm or greater. With these large separations, a single myosin molecule can't simultaneously bind to both filaments. There are then 75 measurements, with separations ranging from 50-250 nm.

To simulate these experiments, we started with a vesicle having a single motor bound to an actin filament. We then put the crossing filament 2.0 µm away from the initial attachment point, and added random noise from a uniform distribution with a maximum of 1 and minimum of −1

μm, so that the crossing filament was initially 1 - 3 μm from the initial attachment point. We also randomized the polarity of the intersecting actin filament. Thus, the initial approach angle is dictated by the stochastic motion of the motors that transport the vesicle. Additionally, by placing the crossing filament some distance away from the initial attachment point, we ensure that the attachment/detachment of motors has reached steady-state.

Each simulated encounter with an intersecting actin filament consisted of 300 chemical reactions (i.e. forward motors steps, backward steps, attachments and/or detachments). For each simulation, we recorded whether the motion terminated (defined as a detachment within one vesicle diameter of the intersection), the vesicle continued straight (defined as all motors attached to the original actin filament, and the vesicle greater than one diameter from the intersection), the vesicle turned (defined as all motors attached to the new actin filament, and the vesicle greater than one diameter from the intersection), the tug of war did not finish (if motors were bound to both filaments), or the simulation was inconclusive (all other cases, e.g. detachment prior to the vesicle reaching the intersection). We simulated 75 such encounters with an intersecting filament, in order to match the number of experimental measurements. Once all 75 simulations were complete, we re-ran all simulations where the tug of war did not finish or the simulation was inconclusive until we got a terminate, turn or straight result.

In the simulations, we can control the separation between the filaments, but we cannot control the approach angle of the vesicle, since it is determined by the stochastic motion of the motors. Thus, in order to approximate the approach angles observed in the experiments, we performed each of the 75 simulations, described above, five times. From these 375 simulations, we kept the

75 whose filament separations matched and whose approach angle were closest to the experimentally observed values.

We then repeated this process six times (a total of 2,250 individual simulations). Based on the vesicle's approach angle and the separation between the actin filaments, we determined whether a myosin molecule would have access to the crossing filament. When a myosin molecule could bind to the crossing filament, we determined whether the vesicle went straight, turned or terminated, as we did for the experimental measurements. In our simulations (Supplementary Figure 12B), we found 61±5% straight (mean±s.d., c.f. 62% in the experiments), 33±5% turn (c.f. 33% in the experiments), and 6±4% terminate (c.f. 5% in the experiments). Overall, the agreement is reasonable and the simulation results are not significantly different from the experimental measurements ($p>0.05$, $\chi^2$ test).

*3.3.3 Simulated experiments accurately reflect outcome probabilities*

Our six simulated experiments all had similar probabilities of going straight through, turning at, or terminating at an actin intersection, as demonstrated by standard deviations in each of around 5% (giving a s.e.m. of 2%). To further examine whether our simulation results accurately reflect outcome probabilities, we examined all 2,250 simulations and ran an additional 2,000 simulations. Of these 4,250 simulations, 2,910 had filament separations and approach angles where the vesicle was predicted to interact with the crossing filament. For these, 60.0% went straight (c.f. 61±5% for the six simulations), 33.2% turned (c.f. 33±5%) and 7.2% terminated (c.f. 6±4%). Thus, the model predicts that our experimentally observed outcomes accurately reflect the true outcome probabilities to within a few percent.

*3.3.4 Actin polarity does not affect outcome probabilities*

In our experiments, we found that the number of vesicles that turned left at an actin intersection ($n$=16) was almost identical to the number that turned right at an actin intersection ($n$=15). This result suggests that actin polarity does not affect whether a vesicle turns or goes straight through an actin intersection. In our six simulations, we also found no effect of actin polarity on the likelihood of turning. Actin filaments with plus-ends to the left exhibited turns 31.0±8.4% of the time, while actin filaments with plus-ends to the right exhibited turns 33.4±4.5% of the time (differences not significant, $p$>0.05, t-test).

To further investigate whether filament polarity had an effect on the outcome of a simulation (i.e. straight, turn, or terminate), we looked at the outcome of all 4,250 simulated trajectories. For all simulations where the plus-ends were to the left, and where a myosin molecule would have access to the crossing filament, the outcomes were straight 59.1%, turn 33.2%, terminate 7.8% ($n$ = 1,378). For all simulations where the plus-ends were to the right, and where a myosin molecule would have access to the crossing filament, the outcomes were straight 60.1%, turn 33.2%, terminate 6.8% ($n$ = 1,532). Given the similarity of these percentages, we conclude that actin filament polarity has little effect on simulation outcome.

*3.4 Sensitivity analysis*

The parameters used in our simulations were $\ell$ = 50 nm, $p_s$ = 0.22, $k_a$ = 2.4 $s^{-1}$ and $\kappa_\theta$ = 0.25 pNnm/rad. We performed a sensitivity analysis to determine how our results depend on these parameters.

*3.4.1 Myosin rest length and spiral pitch*

Our best estimate for myosin's rest length, $\ell$, is 50 nm. However, we expect that this value can vary depending on myosin's state (i.e. whether one or two heads are bound). Further, although myosin's full reach is likely around 50 nm, it might be reasonable to position the hinge in our mechanical model of myosin (Supplementary Figure 8B) at the junction of myosin's two lever arms, decreasing $\ell$ to 35 nm (e.g.[4]). We therefore performed simulations with $\ell = 35$ nm.

For each of these simulated data sets, we performed 75 simulations, with filament gaps matching a subset of our experimental measurements. For these 75 simulations, we determined the proportion of trajectories that went straight, turned and terminated, for trajectories with an approach angle where interaction was predicted. We repeated this process 10 times, and found the average and standard deviation.

At the initiation of our simulations, the crossing actin filament was randomly placed between 1 and 3 μm away from the vesicle. This variation of 2 μm ensures that, given a spiral pitch of ~ 2 μm we uniformly sample all possible approach angles. However, since the motors are stochastic, even when the average spiral pitch is 2 μm, some vesicles can follow spirals with a longer pitch over a 1-3 μm distance. Thus, to determine whether a bias in approach angle affected our results, we performed simulations with a shorter ($p_s = 0.33$, $P_s = 1.5$ μm) spiral pitch.

Regardless of spiral pitch and myosin length, all simulations gave similar results (Supplementary Figure 13A). Thus, errors in our estimates of these values have a minimal effect on our conclusions.

*3.4.2. Myosin stiffness and attachment rate*

The model has two parameters, $k_a$ and $\kappa_\theta$, that were unknown. We used $k_a$ = 2.4 $s^{-1}$ and $\kappa_\theta$ = 0.25 pN nm/rad in our simulations. To determine how these variables affect our conclusions, we performed a sensitivity analysis. Because a shorter spiral pitch does not affect simulation outcomes, in these simulations we used $p_s$ = 0.33, giving a spiral pitch of $P_s$ = 1.5 µm, in order to ensure a uniform sampling of approach angles.

We examined 17 different combinations of $k_a$ and $\kappa_\theta$. For each parameter set, we performed ten simulations of our experiments, as described above, and, when the vesicle had an approach angle where interaction was predicted (Supplementary Figure 4, Interaction geometries of Supplementary Figure 12A), we recorded trajectories that went straight, turned or terminated. The accumulated results of these ten simulations was then compared to data, and we determined whether the simulations were different from ($p<0.05$, $\chi^2$ test) or not different from ($p>0.05$, $\chi^2$ test) the measurements. This data met all requirements for analysis by $\chi^2$ test. These simulations allow us to identify a region of parameter space where simulation and experiment are not significantly different (yellow region in Supplementary Figure 13, bottom right). Note that, when $k_a$ gets too large, we expect that more than three motors will be bound. This provides an upper-bound to $k_a$.

Generally, we find that turning probability increases with attachment rate, but rapidly saturates at around =0.5 - 1 $s^{-1}$. For example, when $\kappa_\theta$ is held fixed at 0.25 pNnm/rad, increasing the attachment rate ($k_a$ = 0.4, 0.8, 1.6, 2.4, and 3.2 $s^{-1}$) decreases the termination probability at the expense of turning, with little difference in the probability of going straight (see Supplementary

Figure 13B, left). For this value of $\kappa_\theta$, only the lowest attachment rate was significantly different from the measurements.

We also find that turning probability decreases with torsional stiffness. For example, when $k_a$ is held fixed at 2.4 $s^{-1}$, increasing the angular stiffness ($\kappa_\theta$ = 0.0625, 0.25, 1, 2, 4 pNnm/rad) leads to a decrease in turning probability, with the probability of going straight increasing and the probability of terminating remaining roughly constant (see Supplementary Figure 13B, top). For this value of $k_a$, only the highest torsional stiffness was significantly different from the measurements.

### *3.4.3 Summary of sensitivity analysis*

The sensitivity analysis demonstrates two main points. First, the qualitative result of our experiments and simulations, that motor teams prefer to go straight through 3D actin intersections, is generic. For all parameter combinations we tested, straight trajectories were always most frequent. Second, there is quantitative agreement between our simulations and experiments for a wide range of parameter values. In fact, all simulations with an attachment rate higher than $k_a$ = 1 $s^{-1}$ and a stiffness lower than $\kappa_\theta$ = 2.5 pNnm/radian were not different from the experiments. Thus, agreement between model and experiment is robust, and is unlikely to be affected by model assumptions.

**Supplementary References:**


1. Trybus, K. M. Myosin V from head to tail. *Cell Mol Life Sci* **65**, 1378-1389, doi:10.1007/s00018-008-7507-6 (2008).
2. Tolic-Norrelykke, S. F. *et al.* Calibration of optical tweezers with positional detection in the back focal plane. *Rev Sci Instrum* **77**, doi:Artn 10310110.1063/1.2356852 (2006).
3. Ali, M. Y. *et al.* Myosin Va maneuvers through actin intersections and diffuses along microtubules. *P Natl Acad Sci USA* **104**, 4332-4336, doi:10.1073/pnas.0611471104 (2007).
4. Vilfan, A. Elastic lever-arm model for myosin V. *Biophys J* **88**, 3792-3805, doi:10.1529/biophysj.104.046763 (2005).
5. Nelson, S. R., Trybus, K. M. & Warshaw, D. M. Motor coupling through lipid membranes enhances transport velocities for ensembles of myosin Va. *P Natl Acad Sci USA* **111**, E3986-E3995, doi:10.1073/pnas.1406535111 (2014).
6. Kad, N. M., Trybus, K. M. & Warshaw, D. M. Load and P(i) control flux through the branched kinetic cycle of myosin V. *J Biol Chem* **283**, 17477-17484, doi:10.1074/jbc.M800539200 (2008).
7. Walcott, S., Turner, M., Woodward, M. & Debold, E. P. Direct Observation of Phosphate Inhibiting the Force-Generating Capacity of a Mini-Ensemble of Myosin Molecules. *Biophys J* **106**, 156a-156a (2014).
8. Gillespie, D. T. Exact Stochastic Simulation of Coupled Chemical-Reactions. *J Phys Chem-Us* **81**, 2340-2361, doi:Doi 10.1021/J100540a008 (1977).
9. Ali, M. Y. *et al.* Myosin V is a left-handed spiral motor on the right-handed actin helix. *Nat Struct Biol* **9**, 464-467, doi:10.1038/nsb803 (2002).
